# RPA–ssDNA co-phase separation facilitates RAD51 enrichment during homologous recombination

**DOI:** 10.1093/nar/gkag586

**Published:** 2026-06-11

**Authors:** Yanan Li, Yi Zhao, Yinghong Chen, Teng Wang, Lulu Bi, Yanling Bao, Lishuang Chen, Xia Zhang, Bingkai Cheng, Meng Hu, Shengli Jing, Chao Liu, Wei Li, Bo Sun

**Affiliations:** College of Life Sciences, Xinyang Normal University, Xinyang, Henan 464000, China; School of Life Science and Technology, ShanghaiTech University, Shanghai 201210, China; School of Life Science and Technology, ShanghaiTech University, Shanghai 201210, China; Guangzhou Women and Children’s Medical Center, Guangzhou Medical University, Guangzhou,Guangdong 510623,China; Department of Basic Medical Sciences, Shantou University Medical College, Shantou, Guangdong 515041, China; School of Life Science and Technology, ShanghaiTech University, Shanghai 201210, China; Department of Medical Laboratory, Jining No. 1 People’s Hospital, Jining, Shandong 272011, China; School of Life Science and Technology, ShanghaiTech University, Shanghai 201210, China; Institutes of Biomedical Sciences, Shanghai Medical College of Fudan University, Shanghai 200032, China; School of Life Science and Technology, ShanghaiTech University, Shanghai 201210, China; School of Life Science and Technology, ShanghaiTech University, Shanghai 201210, China; School of Life Science and Technology, ShanghaiTech University, Shanghai 201210, China; School of Life Science and Technology, ShanghaiTech University, Shanghai 201210, China; School of Life Science and Technology, ShanghaiTech University, Shanghai 201210, China; College of Life Sciences, Xinyang Normal University, Xinyang, Henan 464000, China; Guangzhou Women and Children’s Medical Center, Guangzhou Medical University, Guangzhou,Guangdong 510623,China; Guangzhou Women and Children’s Medical Center, Guangzhou Medical University, Guangzhou,Guangdong 510623,China; School of Life Science and Technology, ShanghaiTech University, Shanghai 201210, China

## Abstract

Homologous recombination (HR) is pivotal for the maintenance of genome integrity. During HR, replacing replication protein A (RPA) with the recombinase RAD51 on single-stranded DNA (ssDNA) is crucial in forming the presynaptic complex for homology search. However, how RAD51 identifies legitimate RPA-coated ssDNA involved in HR to prepare for the replacement remains elusive. Here, we develop an innovative approach to generate ssDNA for the single-molecule measurements of this transaction. We find that human RPA can undergo phase separation and co-condense with coated ssDNA, a process primarily mediated by the N-terminal DNA-binding domain of RPA70 (RPA70N). The resulting RPA–ssDNA co-condensates form stable nucleoprotein assemblies that withstand disruptive forces of tens of piconewtons. Intriguingly, both *in vitro* and *in vivo* evidence indicates that these co-condensates act as hubs for the local enrichment of RAD51. Consequently, deletion or sequestration of RPA70N readily suppresses the phase separation ability of RPA, reduces RAD51 enrichment, and ultimately compromises DNA double-strand break (DSB) repair. Our work defines the key determinants underlying RPA-mediated recombinase enrichment during the early stage of HR-directed DSB repair.

## Graphical Abstract

**Graphical Abstract:**
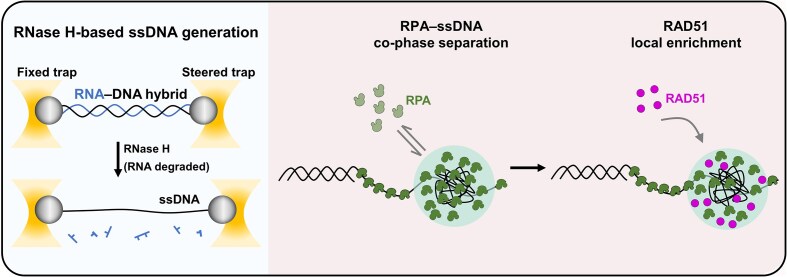

## Introduction

Homologous recombination (HR) is a genetic process that exchanges genetic information between two similar or identical nucleic acid molecules [1, 2]. This recombination process is responsible for the repair of DNA double-strand breaks (DSBs), the rescue of stalled or collapsed replication forks, and the crossing-over of chromosomes during meiosis [2–5]. The physiological significance of HR is underscored by the apparent genomic instability in HR-deficient cells and their association with cancer predisposition and cancer-prone diseases [4, 6–8]. Essential HR genes thus act as genomic caretakers, and their variations have been exploited as targets for cancer therapies [9, 10]. An in-depth understanding of the molecular mechanisms underlying HR is crucial for developing potential therapeutic strategies.

HR is a complex multistep process that involves coordinating several DNA-interacting proteins [11–13]. A central step in HR is the exchange of strands between a single-stranded DNA (ssDNA) molecule and a homologous double-stranded DNA (dsDNA) molecule, which is catalyzed by the recombinases, the RecA/RAD51 family of ATPases [2, 4, 13, 14]. In eukaryotes, the recombinase RAD51 forms extended helical filaments on ssDNA, and this nucleoprotein filament, termed presynaptic complex, is the functional unit to search and invade homologous DNA for pairing [4, 13, 15]. However, following the initial stage of HR, where helicases and nucleases generate long 3′ ssDNA overhangs, replication protein A (RPA), rather than RAD51, is the first responder in eukaryotes that binds ssDNA in a sequence-independent manner with a subnanomolar affinity (*K*_D_ ∼ 10^−10^ M) [16–19]. As a ssDNA-binding protein, RPA stabilizes ssDNA structures and protects them from nucleolytic degradation. Thereby, RAD51 has to replace ssDNA-bound RPA to assemble the presynaptic complex. However, RPA is an abundant protein in cells and can rapidly associate with almost all ssDNA generated in various nucleic acid metabolic processes [17, 20–22]. These facts raise a question of how RAD51 identifies the particular ssDNA-bound RPA complex that needs to be legitimately replaced for HR. Moreover, the RPA-to-RAD51 switch is kinetically unfavorable because RAD51 has a much lower ssDNA affinity (*K*_D_ ∼ 10^−6^ M). In addition to using mediator proteins, such as BRCA2, to alleviate the kinetic barrier, the multiple and transient interactions of RPA with ssDNA have been acknowledged to contribute to the dynamic turnover [4, 23–26]. Structural, biochemical, and single-molecule studies have been conducted to thoroughly comprehend the dynamic interactions between RPA and ssDNA [16, 27–31].

RPA is a heterotrimeric protein complex with three tightly associated subunits: RPA70, RPA32, and RPA14 [32–34]. Structurally, the human RPA complex contains six oligonucleotide/oligosaccharide-binding-fold domains (also termed as DNA-binding domains, DBDs) that are connected by flexible linkers of varying lengths [16, 33, 35]. Four of the DBDs (DBD-A, DBD-B, DBD-C, and DBD-F) are located in the largest RPA70 subunit, and DBD-D and DBD-E reside on the mid-sized RPA32 subunit and the smallest RPA14 subunit, respectively [20, 33, 36–38]. With distinct binding affinities, these six DBDs can adopt multiple conformations and bind ssDNA in different modes dependent on ssDNA length [17, 36, 39, 40]. RPA can also diffuse along ssDNA, which allows for the unwinding of DNA secondary structures [41–44]. The binding flexibility and mobility of RPA enable dynamic exchange with other ssDNA-binding proteins [17, 40, 45, 46]. Beyond DBD-D, the RPA32 subunit contains an N-terminal phosphorylation motif (RPA32-PM) and a C-terminal winged helix domain of RPA32 (RPA32-WH). Within the RPA complex, DBD-F (also known as RPA70N) and RPA32-WH (also known as RPA32C) serve as two dedicated modules that facilitate interaction and coordination with over three dozen proteins vital for the HR process and DNA damage-checking signaling, such as p53 [16, 20, 27, 30, 38, 47–49]. Besides stoichiometric binding to ssDNA, RPA has recently been demonstrated to assemble into phase-separated condensates with ssDNA. This aggregation process has been attributed to the intrinsically disordered region (IDR) of RPA32-PM [50]. RPA phase separation has been linked to telomere maintenance by alternative lengthening of telomeres in cancer [50]. However, our knowledge of the potential regulatory roles of RPA phase separation in HR is still limited.

In this study, we developed an RNase H-based approach for ssDNA production that significantly simplifies single-molecule visualization and measurement of dynamic protein–ssDNA interactions. We found that RPA can undergo phase separation on a single ssDNA molecule to form condensates, leading to simultaneous ssDNA condensation. Surprisingly, the RPA–ssDNA co-condensate constitutes a stable nucleoprotein structure that can withstand a disruptive force of up to 200 pN. Moreover, phase-separated RPA on ssDNA acts as a clustering platform that facilitates the local enrichment of RAD51, thereby increasing its effective local concentration and priming the system for subsequent RPA replacement. Experiments with truncated RPA variants verified that the DBD-F of RPA70 (hereafter referred to as RPA70N) is indispensable in mediating RPA phase separation. Consequently, depletion of RPA70N led to impaired RPA phase separation and thus retarded RAD51 enrichment, which ultimately repressed DSB repair. Overall, our findings provide a dynamic perspective on ssDNA regulation by phase-separated RPA and support a model in which RPA condensates promote recombinase enrichment during the early stages of HR.

## Materials and methods

### Protein preparation

#### RPA and its variants

The plasmid encoding enhanced green fluorescent protein (eGFP)-tagged full-length (FL) human RPA or its variants, including RPA-FL-eGFP, RPA-Δ32C-eGFP (deletion of amino acids 171–270 in the RPA32 subunit), RPA-Δ32N-eGFP (deletion of amino acids 1–44 in the RPA32 subunit), and RPA-Δ70N-eGFP (deletion of amino acids 1–181 in the RPA70 subunit), was constructed by synthesizing the open reading frames of the three subunits and inserting them into the pET28a vector. This construct (pET28a-RPA-FL-eGFP) encodes an N-terminal His_6_-tagged RPA70 with a C-terminal eGFP tag, along with RPA32 and RPA14. The pET28a-RPA-FL-eGFP plasmid serves as the base vector for constructing variants by polymerase chain reaction (PCR). Co-expression of the subunits was carried out in *Escherichia coli* BL21 (DE3) cells (TransGen). RPA-FL-eGFP was purified following a previously described protocol [51]. Briefly, the expression plasmid was transformed into BL21 (DE3) cells, and expression was induced by adding 1 mM isopropyl-1-thio-D-galactopyranoside (IPTG) at 18°C for 16 h. Cells were harvested by centrifugation, resuspended in lysis buffer containing 20 mM Tris–HCl (pH 7.5), 500 mM NaCl, 10 mM imidazole, 5% glycerol, and 1 mM phenylmethylsulfonyl fluoride, and disrupted by passage through a homogenizer three times at ∼800 bar. The lysate was subsequently ultracentrifuged at 11 000 × *g* for 35 min. The supernatant was applied to Ni-NTA resin and washed with increasing concentrations of imidazole (10, 30, 50, and 300 mM) in lysis buffer. RPA-FL-eGFP was eluted using a buffer containing 50 mM Tris–HCl (pH 7.5), 500 mM NaCl, 300 mM imidazole, and 5% glycerol. Further protein purification and ssDNA removal were achieved by chromatography on a 5 ml HiTrap Heparin HP column. Proteins were eluted with a linear NaCl gradient from 150 to 1 000 mM over 15 column volumes. ssDNA-free RPA bound efficiently to the column and was subsequently eluted at 200 mM NaCI. The sample was then subjected to size-exclusion chromatography using a Superdex 200 Increase 10/300 column (GE Healthcare) (Supplementary Fig. S1A). Finally, the protein was buffer-exchanged into storage buffer containing 50 mM Tris–HCl (pH 7.5), 500 mM NaCl, 10% glycerol, and stored at −80°C. Other RPA proteins were expressed and purified similarly. Other RPA variants were expressed and purified using the same protocol, and their ssDNA-binding activities were subsequently assessed (Supplementary Fig. S1A and B).

#### RAD51

The open reading frame of human RAD51 was subcloned into the pET28a vector (Novagen) between the NcoI and SalI restriction sites. Cysteine-to-serine variants (RAD51-C319S) were generated using the QuikChange site-directed mutagenesis method to enable fluorescent labeling. The expression plasmid was transformed into *E. coli* BL21 (DE3) cells, which were cultured and induced with 1 mM IPTG at 18°C for 16 h. Cells were harvested by centrifugation and resuspended in lysis buffer containing 50 mM Tris–HCl (pH 7.5), 300 mM NaCl, 10 mM imidazole, 5% glycerol, and 1 mM phenylmethylsulfonyl fluoride. Cell lysis was performed by passing the suspension through a homogenizer three times at ∼800 bar, followed by ultracentrifugation at 11 000 × *g* for 35 min. The supernatant was applied to Ni-NTA resin and subjected to stepwise washing with imidazole concentrations of 10, 30, 50, 100, and 300 mM in the lysis buffer. RAD51 was eluted at 300 mM imidazole. Further purification was achieved using a 5 ml HiTrap Heparin HP column. Further protein purification and DNA removal were performed using a 5 ml HiTrap Heparin HP column. Proteins were eluted with a linear NaCl gradient from 150 to 1 000 mM over 15 column volumes. DNA-free RAD51 bound efficiently to the column and was subsequently eluted at 200 mM NaCI. For fluorescent labeling, purified RAD51 (1 mg/ml) was treated with 20 mM DTT and incubated on ice for 30 min. The sample was then buffer-exchanged into labeling buffer containing 50 mM MOPS-HCl (pH 7.0), 300 mM KCl, 1 mM ethylenediaminetetraacetic acid (EDTA), and 10% glycerol, which had been degassed with argon for 30 min. Maleimide-coupled Cy3 dye (Abcam) was resuspended in labeling buffer and added to the protein sample at a five-fold molar excess. The reaction mixture was incubated on ice for 30 min and quenched by adding 20 mM DTT, followed by an additional 30-min incubation on ice. Excess dye was removed by buffer exchange into a storage buffer containing 300 mM KCl, 50 mM Tris–HCl (pH 7.5), 1 mM EDTA, 2 mM DTT, and 10% glycerol (Supplementary Fig. S1C).

#### RNase H and p53

The human p53 (R175H) was purchased from YEASEN (Product No: 93008ES20) [48]. The *E. coli*. RNase H was purchased from NEB (Product No: M0297S).

### Preparation of RNA–DNA hybrid templates

**Figure 1.**
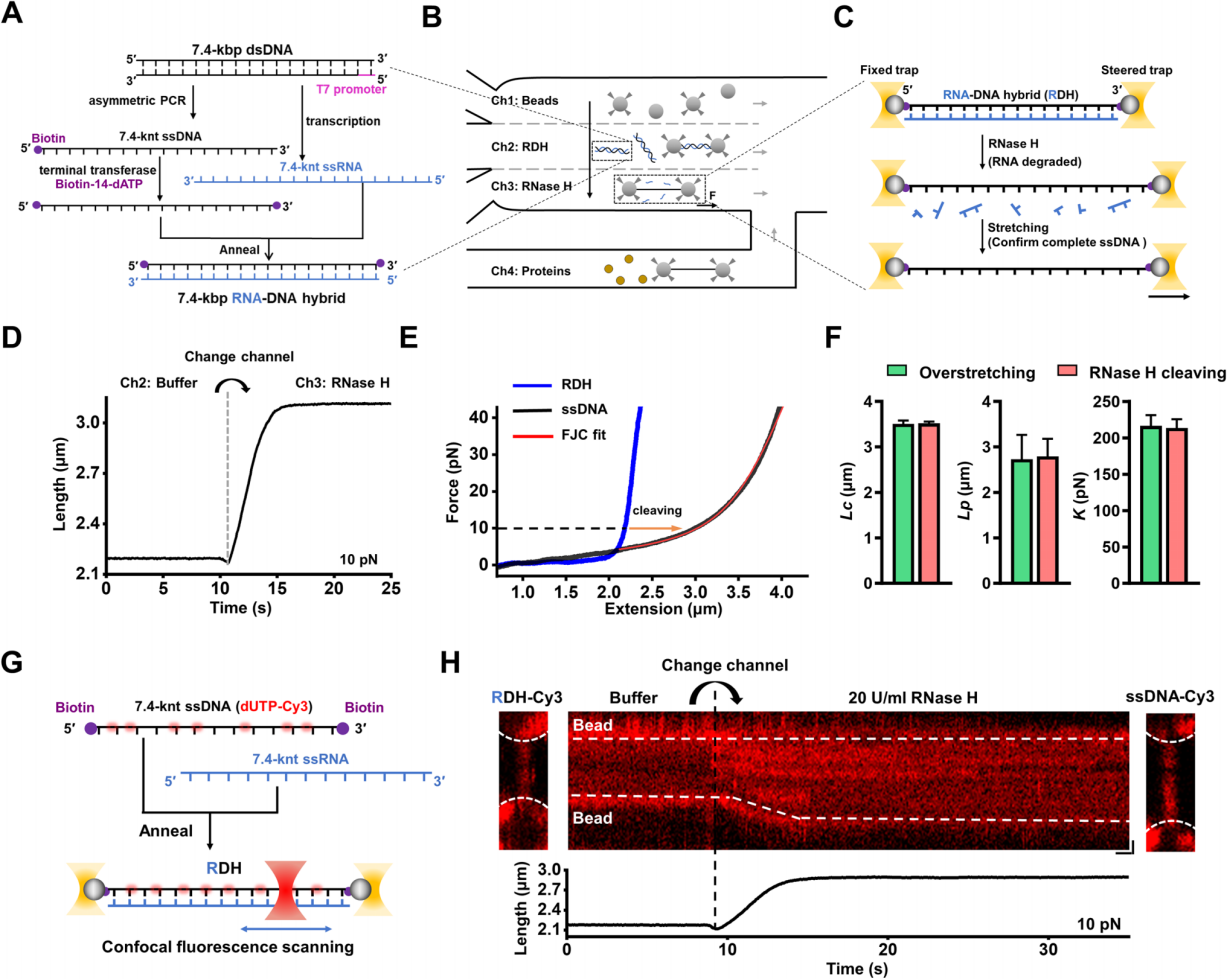
An RNase H-based approach to generate ssDNA in the OT assay. (**A**) Schematic of the construction of the RDHs template used to produce ssDNA in OT assays. A dsDNA segment containing a T7 promoter is PCR-amplified from the lambda DNA. The ssDNA is generated via asymmetric PCR amplification using a biotin-labeled primer. The 3′ end of the ssDNA is biotinylated using biotinylated dATP (APExBIO) and terminal transferase. The ssRNA is transcribed from the dsDNA containing the T7 promoter. The resulting 7.4-kbp RDH template is formed by annealing the ssDNA with its complementary ssRNA. (**B**) Schematic of the experimental configuration. An RDH tether is initially formed in channels 1–2 containing streptavidin-coated beads and biotinylated RDH, respectively. The tether is then moved to channel 3, which contains RNase H, an enzyme that degrades the RNA strand in the hybrid to produce ssDNA. Finally, the ssDNA tether is transferred to channel 4, which contains only the buffer or protein of interest. (**C**) A zoomed-in view of RNase H degrading the hybrid. In channel 3, RNase H degrades the RNA strand of the hybrid under a force of 10 pN. After degradation, an ssDNA molecule is verified by force–extension relationship. (**D**) The RDH template length as a function of time in the absence and presence of RNase H (20 U/ml). (**E**) The force–extension relationship of the RDH template (blue) and the ssDNA template (gray) produced by RNase H degrading the RDH in phosphate buffered saline (PBS) buffer. The force–extension curves of the ssDNA are fitted using the freely jointed chain (FJC) model (red). (**F**) The contour length (*Lc*), persistence length (*Lp*), and stretching modulus (***K***) of ssDNA prepared using two different techniques: RDH overstretching and RNase H cleavage. The data are shown in mean ± SD, with *n* = 6. (**G**) Schematic of the construction of fluorescently labeled ssDNA using dUTP-Cy3 for the confocal-combined OT assays. (**H**) A representative kymograph showing that ssDNA-Cy3 is generated by RNase H degrading dUTP-Cy3 labeled RDH. The corresponding hybrid length of the examined molecule is displayed below the kymographs. Scale bars, 1 s (horizontal), 0.5 μm (vertical).

The RNA–DNA hybrid (RDH) templates used in the optical tweezers (OT) assays were prepared as follows (Fig. 1A and Supplementary Table S1). The template consists of biotinylated ssDNA on both ends, paired with a ssRNA. A dsDNA segment was PCR-amplified from lambda DNA (Thermo). A second asymmetric PCR reaction was performed to generate the ssDNA using this dsDNA segment and a biotin-labeled primer. For the fluorescence labeling of the ssDNA, extra dUTP-Cy3 was added in the second asymmetric PCR reaction. The resulting dsDNA and ssDNA were separated by agarose gel electrophoresis and purified using the GeneJET Gel Extraction Kit (Thermo). The 3′ end of the ssDNA was biotinylated using biotin-dATP (Invitrogen) and terminal transferase (NEB). A dsDNA fragment, including a T7 promoter at the 5′ end (T7 DNA), was PCR-amplified from lambda DNA to construct the ssRNA. The ssRNA was then transcribed from the T7 DNA template. Finally, the RDH templates were formed by annealing the ssDNA and ssRNA in TE buffer. The length of the hybrids depends on the needs of the experiments.

To better mimic physiologically relevant DNA structures generated during HR, we designed an RNA–DNA/dsDNA hybrid template to produce ss/dsDNA substrates (Supplementary Fig. S2). A 12.3-kbp dsDNA fragment was PCR-amplified from λ DNA. A 12.3-knt ssDNA was generated by asymmetric PCR using a biotin-labeled primer, followed by 3′-end biotinylation with biotin-dATP (APExBIO) and terminal transferase. The 12.3-kbp dsDNA was further divided into 10- and 2.3-kbp fragments. A 10-knt ssRNA was transcribed *in vitro* from the dsDNA fragment containing a T7 promoter, and a 2.3-knt ssDNA was generated by asymmetric PCR. The final 12.3-kbp RNA–DNA/dsDNA hybrid substrate was assembled by annealing the 12.3-knt ssDNA with the complementary 10- and 2.3-knt ssDNA fragments.

### Single-molecule optical tweezers assay and data analysis

Single-molecule OT assays were performed at 25°C on an instrument combining three-color confocal fluorescence microscopy with dual optical traps (LUMICKS C-trap, Netherlands) [52–54]. The assay initially captured an RDH molecule between two streptavidin-coated polystyrene beads (1.76 μm in diameter, Spherotech). This hybrid tether was then transferred to the RNase H channel (20 U/ml), where RNA in the hybrid was entirely degraded by RNase H to produce ssDNA. Experiments involving different proteins, such as RPA-eGFP and its variants, were conducted in a reaction buffer containing 25 mM Tris–HCl (pH 7.5), 100 mM NaCl, 1 mM MgCl_2_, 0.1 mg/ml bovine serum albumin (BSA), and 3 mM DTT, with variations depending on the specific experiment. For RAD51 experiments, the reaction buffer consisted of 25 mM Tris–HCl (pH 7.5), 100 mM KCl, 2 mM CaCl_2_, 0.1 mg/ml BSA, and 1 mM DTT. Imaging of RPA-eGFP, RAD51-Cy3, and Cy3-labeled ssDNA was performed using 488- and 532 nm excitation lasers, respectively. The confocal pixel size was set to 50 or 75 nm, with a pixel dwell time of 0.2 or 1 ms. Single-molecule data analysis was conducted using custom software from LUMICKS. Pseudocolor adjustments for enhanced signal contrast were applied in Fiji. For fluorescence signal quantification, total pixel intensities in the ssDNA region were summed for each frame using Fiji.

### *In vitro* phase separation assay

For the *in vitro* phase separation assay, 10 μM RPA-FL-eGFP and its truncated variants were mixed with a buffer containing 1.7 μM 33-nt ssDNA-Cy5, 25 mM Tris–HCl (pH 7.5), 100 mM NaCl, 3 mM DTT, 1 mM MgCl_2_, and 5% (w/v) PEG-8000, respectively. Unless otherwise stated, the mixture was incubated at room temperature for 20 min before imaging. In the *in vitro* phase separation experiment involving sequestration of RPA70N by p53, 10 μM p53 was pre-incubated with RPA-eGFP in the buffer, omitting PEG-8000 and ssDNA, for 2 h. After incubation, 5% PEG-8000 and 1.7 μM 33-nt ssDNA-Cy5 were added to the samples before imaging. In the *in vitro* phase separation experiment of co-phase separation between RPA and RAD51, 5 μM RAD51-Cy3 was added to the samples. For imaging, 10 μl of each sample was pipetted onto a glass-bottom dish, and images were captured using a 63× oil immersion objective on a Leica SP8 confocal microscope. The size of the condensates was quantified using the “Analyze Particles” function in Fiji software. Data analysis and graphing were performed using GraphPad Prism.

### Fluorescence recovery after photobleaching assay

The *in vitro* fluorescence recovery after photobleaching (FRAP) experiments were conducted using the FRAP module of the Leica SP8 confocal microscopy system. Photobleaching was performed using three laser beams with wavelengths of 480, 488, and 497 nm, focusing the photobleaching on a circular region of interest (ROI) with 100% laser power. Time-lapse images were captured to monitor the fluorescence recovery. Fluorescence intensity measurements were performed using Fiji software, with background intensity subtracted. Data are presented relative to the pre-bleach time points. GraphPad Prism was used to visualize and analyze the FRAP data.

The *in vivo* FRAP experiments were conducted using the stimulation module of the Nikon AX R microscope (Tokyo, Japan). Fluorescence intensity was recorded using NIS-Elements software. RPA–ssDNA condensates were selected as a circular ROI to quantify the phase separation ability of RPA–ssDNA accurately. Fluorescence intensity was recorded for one frame before bleaching, and recovery was monitored at a rate of one frame every 2 s over 100 s. Background intensity was simultaneously recorded and subtracted during the analysis of the FRAP data. GraphPad Prism was employed to visualize and analyze the FRAP data.

### Cell culture, transfection, and knockdown

Human osteosarcoma (U2OS) cells were obtained from the American Type Culture Collection and cultured at 37°C under standard cell culture conditions (humidified atmosphere, 5% CO_2_) in McCoy’s 5A modified medium supplemented with 10% fetal bovine serum (Gibco) and 1% penicillin-streptomycin antibiotics. Where indicated, cells were treated with 4 μM camptothecin (CPT) for 5 h, followed by washing with PBS, permeabilization, fixation, and subsequent processing as described. U2OS cells were transfected with the relevant plasmid DNA (~0.5 µg per 10^5^ cells) using 2 µl of LipoMax DNA transfection reagent (Sudgen, 32010) and incubated for 24 h. For knockdown experiments, siRNA duplexes targeting RPA1 (25 nM siRNA per 10^5^ cells) were transfected using Lipofectamine™ RNAiMAX reagent (Invitrogen, 13778030) alongside nontargeting control small interfering RNA (siRNA) duplexes. The sequences of siRNA duplexes are provided in Supplementary Table S2, with nontargeting siRNA serving as the negative control. Cells were harvested 2.5 days post-transfection for fluorescence microscopy and lysate preparation.

### Immunofluorescence

Cells were seeded onto coverslips in six-well plates 18 h prior to treatment. For RAD51 and RPA staining, cells were pre-extracted on ice for 10 min using a pre-extraction buffer containing 20 mM HEPES (pH 7.4), 20 mM NaCl, 5 mM MgCl_2_, 0.5% NP-40, 1 mM DTT, and a protease inhibitor cocktail. Following pre-extraction, cells were fixed with 4% paraformaldehyde (PFA) for 10 min. Cells were then washed three times with PBS and permeabilized for 30 min in 0.5% Triton X-100/PBS. Before staining, cells were blocked with 5% BSA for 3 h at room temperature. Primary antibody incubation was performed overnight at 4°C. After three PBS washes, cells were incubated with secondary antibodies for 1.5 h at room temperature and stained with 4′,6-diamidino-2-phenylindole (DAPI) for 3 min. Finally, cells were washed three more times with PBS, mounted onto glass slides, and observed using confocal microscopy.

### Immunoblotting

Cells were harvested and lysed in cold RIPA buffer (Solarbio, R0010) supplemented with 1 mM phenylmethylsulfonyl fluoride and 1× protease inhibitor cocktail (Roche, 04693132001) on ice for 30 min. The lysates were centrifuged at 13 000 × *g* for 10 min, and the resulting supernatant was mixed with 2× loading buffer containing 1% sodium dodecyl sulfate (SDS). The mixture was boiled at 95°C for 10 min to prepare for subsequent immunoblotting. Protein lysates were separated by SDS–polyacrylamide gel electrophoresis under reducing conditions and transferred onto nitrocellulose membranes using a semi-dry transfer method. Membranes were incubated with primary antibodies, followed by peroxidase-conjugated secondary antibodies. The blots were visualized using the Touch Imager Pro Imaging System (e-BLOT Life Science, China).

### Antibodies

The following primary antibodies were used: mouse monoclonal anti-phospho-Histone H2A.X (Ser139) antibody (Millipore, Cat# 05-636, 1:400 dilution), rabbit monoclonal anti-Brdu antibody (MedChemExpress, Cat# HY-P80038, 1:200 dilution), mouse monoclonal anti-RPA70 antibody (MedChemExpress, Cat# HY-P80884, 1:400 or 1000 dilution), rabbit monoclonal anti-Rad51 antibody (Abmart, Cat# T55080, 1:100 dilution), mouse monoclonal anti-GAPDH antibody (Abclonal, Cat# AC003, 1:3000 dilution), and mouse monoclonal anti-GFP antibody (Abmart, Cat# M20004, 1:1000 dilution).

## Results

### An RNase H-based approach to generate ssDNA for single-molecule studies

Single-molecule manipulation techniques, such as OT and magnetic tweezers, have provided a powerful means to decipher important mechanistic details about the dynamics and mechanical properties of the protein–nucleic acid complex [52, 55, 56]. However, due to the inherent flexibility of long ssDNA molecules, they are likely to entangle and/or form intermolecular and intramolecular secondary structures. Thus, preparing ssDNA substrates directly for the single-molecule measurements remains challenging. Current indirect approaches for the construction of ssDNA often employ high mechanical forces (tens of piconewtons) on dsDNA to either peel off one strand of dsDNA or unzip a hairpin DNA structure [52, 57–59]. However, these approaches suffer from markedly low efficacy due to the instability of ssDNA under high forces. Moreover, in addition to ssDNA, the resulting DNA substrates often contain other DNA structures, such as dsDNA, complicating the experimental design and data explanation [57–59]. To overcome these limitations, we developed an RNase H-based approach to generate a few-kilobase-pair (kbp) long ssDNA molecules in OT assays. Briefly, we took advantage of *E. coli* RNase H, which specializes in efficiently eliminating the RNA moiety within RDHs in a timely fashion [54]. To this end, we first constructed a 7.4-kbp RDH template by slowly annealing a two-sided biotinylated ssDNA and its complementary ssRNA (Fig. 1A). After suspending a single RNA–DNA molecule between two optical traps via streptavidin-coated microspheres in a multi-channel flow cell, we immediately transported it to an RNase H-containing channel and maintained a constant force of 10 pN on the tether for tens of seconds for the elimination of the RNA within the hybrid (Fig. 1B–D). At last, we examined the elasticity of the resulting ssDNA to ensure the complete removal of the RNA in the channel containing buffer only (Fig. 1B and E).

To validate the efficacy of this approach, we first recorded the change in the length of a single RDH molecule in real time. Due to the difference in the elastic response between ssDNA and double-stranded hybrid, RNA digestion would cause an increase in the molecule length [54]. Indeed, in the 20 U/ml *E. coli* RNase H channel, the molecule length increased rapidly under 10 pN and reached an unchanged state in <10 s, indicating that the RNA moiety was quickly removed under this condition (Fig. 1D). It is noteworthy that RNase H typically dissociates from the substrate along with the RNA [54]. To further address whether the elimination of the RNA moiety was thorough, we examined the force–extension relationship of the generated ssDNA template. The obtained force–extension curves can be well described by the FJC model [58]. Moreover, the persistence length (*Lp*), contour length (*Lc*), and stretching modulus (*K*) derived from the fitting are comparable with the parameters obtained with the ssDNA molecules generated by the force-induced denaturation of the RDHs (Fig. 1F and Supplementary Fig. S3). Overall, one third of the captured RDH tethers can be converted to ssDNA using this approach. These findings have proved the versatility and effectiveness of our RNase H-based approach for the ssDNA generation.

To visualize the manipulated ssDNA directly, we incorporated dUTP-Cy3 in the asymmetric PCR amplification and annealed the fluorescently labeled ssDNA with complementary ssRNA to construct the hybrid substrate (Fig. 1G). Combining confocal microscopy with OT allowed us to monitor the status of the suspended ssDNA after the RNA elimination by RNase H (Fig. 1H) [54]. Please note that the uneven fluorescence signal along the DNA is due to the inefficient incorporation of dUTP-Cy3 in the ssDNA. Collectively, we developed an RNase H-based approach that significantly simplifies the ssDNA generation for single-molecule manipulation and visualization.

### RPA binding induces regional condensation of an ssDNA molecule

Next, we set out to investigate the dynamic interplay between ssDNA and human RPA at the single-molecule level using the newly developed ssDNA generation method. We first sought to determine whether RPA binding affects the elasticity of ssDNA. After forming a ssDNA tether between one fixed trap and one steered trap. Once the tension on ssDNA was maintained, the contour length of ssDNA was extended by the coated RPA, a phenomenon that is consistent with previous reports (Supplementary Fig. S4A) [60].

**Figure 2.**
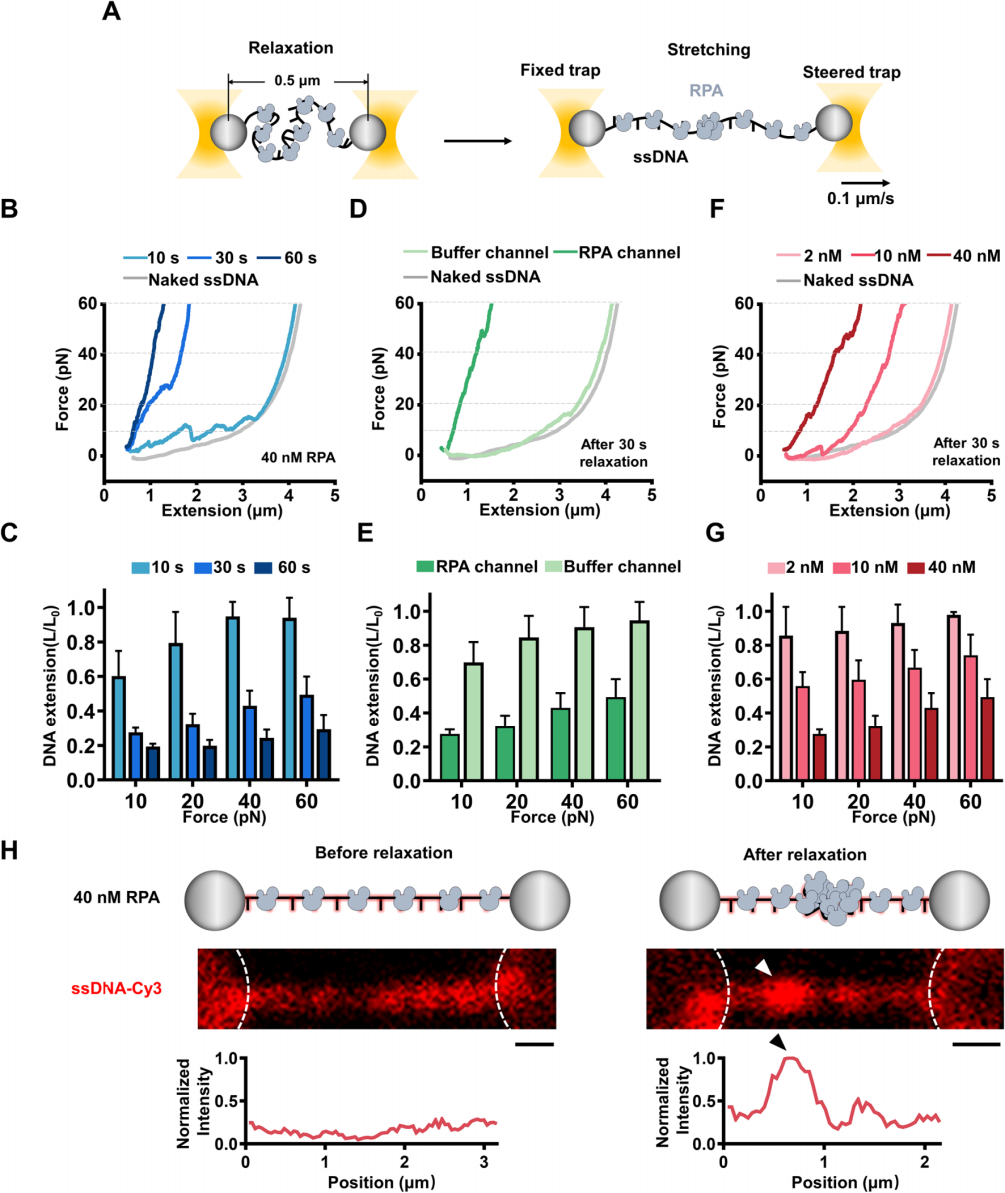
RPA condensing relaxed ssDNA. (**A**) Schematic of the experimental procedures. A suspended ssDNA under a force of 10 pN is incubated in the RPA channel for protein loading. Subsequently, the RPA-bound ssDNA is relaxed by moving the microsphere at the steered trap closer to the fixed trap, maintaining a 0.5 µm separation between the two traps. The RPA-bound, relaxed ssDNA is then stretched by gradually moving the steered trap away from the fixed trap at 0.1 μm/s. (**B**) Representative force–extension curves of RPA-bound ssDNA after relaxation for the indicated time in the presence of 40 nM RPA. The gray line represents the force–extension curve of naked ssDNA. RPA-bound ssDNA templates were stretched after relaxation for 10, 30, and 60 s. (**C**) The normalized DNA extension (*L*/*L*_0_) under different forces obtained from force–extension curves of RPA-bound ssDNA after relaxation for 10 s (*n* = 6), 30 s (*n* = 6), and 60 s (*n* = 7) in the presence of 40 nM RPA. *L*_0_ represents the length of naked ssDNA under the indicated force. Data are presented as mean ± SD. (**D**) Force–extension curves of the relaxed RPA-bound ssDNA in the presence and absence of free RPA and naked ssDNA. (**E**) The normalized relaxed DNA extension (*L*/*L*_0_) under different forces with RPA binding in the presence (*n* = 6) and absence (*n* = 7) of free RPA and naked ssDNA. *L*_0_ represents the length of naked ssDNA. (**F**) Representative force–extension curves of RPA-bound ssDNA after relaxation for 30 s in the presence of 2, 10, and 40 nM RPA. (**G**) The normalized DNA extension (*L*/*L*_0_) under different forces obtained from force–extension curves of RPA-bound ssDNA after relaxation for 30 s in the presence of 2 nM (*n* = 7), 10 nM (*n* = 7), and 40 nM (*n* = 6) RPA. *L*_0_ represents the length of naked ssDNA under the indicated force, respectively. Data are presented in mean ± SD. (**H**) A dUTP-Cy3 labeled RPA-bound ssDNA template (7.4 knt) is suspended between two beads manipulated by two optical traps, with confocal lasers scanning before (left) and after (right) the relaxation and stretching processes, respectively. The corresponding fluorescence profiles of the examined ssDNA are shown below the kymographs. Arrows highlight ssDNA condensation. Scale bar, 0.5 μm.

Considering that ssDNA is not constantly subjected to force under cellular conditions, following RPA binding onto the tensioned ssDNA, we brought the two traps to ~0.5 μm apart (∼0 pN) to allow relaxation of the ssDNA in a channel containing 40 nM RPA-eGFP. After an indicated relaxation period, we immediately moved the steered trap away from the fixed trap at a slow rate of 0.1 μm/s to record the force–extension relationship of the RPA-coated ssDNA (Fig. 2A). Within a relaxation time of 10 s, a “sawtooth” pattern was immediately noticed at a low force range of the force–extension curves. In contrast, these curves largely resemble the naked ssDNA above ∼20 pN (Fig. 2B and C). This finding indicates that the shortening of ssDNA induced by RPA binding was reversed by high forces. However, the force–extension curves obtained after longer relaxation periods (30 or 60 s) revealed significantly shortened ssDNA molecules that could not be recovered even under a high force of 250 pN (Fig. 2B and C; Supplementary Fig. S4B).

To understand how RPA shortens ssDNA, we first examined whether ssDNA-bound RPA is sufficient for condensation. The force–extension relationship of the RPA-coated ssDNA measured in a buffer channel resembles that of the naked ssDNA, corroborating that, in addition to ssDNA-bound RPA, free RPA in solution was also required for the ssDNA shortening (Fig. 2D and E). In support of this, lowering the RPA concentration decreased the extent of ssDNA shortening and even abolished it (Fig. 2F and G). Therefore, ssDNA shortening is an action of multiple RPA proteins and necessitates free RPA in solution. We further used fluorescently labeled ssDNA and revisited the OT assay. By stretching the shortened ssDNA, we noticed a few bright foci within the ssDNA, which were unnoticeable before the shortening. These findings strongly support that RPA-induced ssDNA shortening originates from the local ssDNA condensation (Fig. 2H).

Altogether, a high amount of RPA proteins can regionally condense coated ssDNA and maintain a stable nucleoprotein complex.

### RPA70N is indispensable in RPA-induced ssDNA condensation

**Figure 3.**
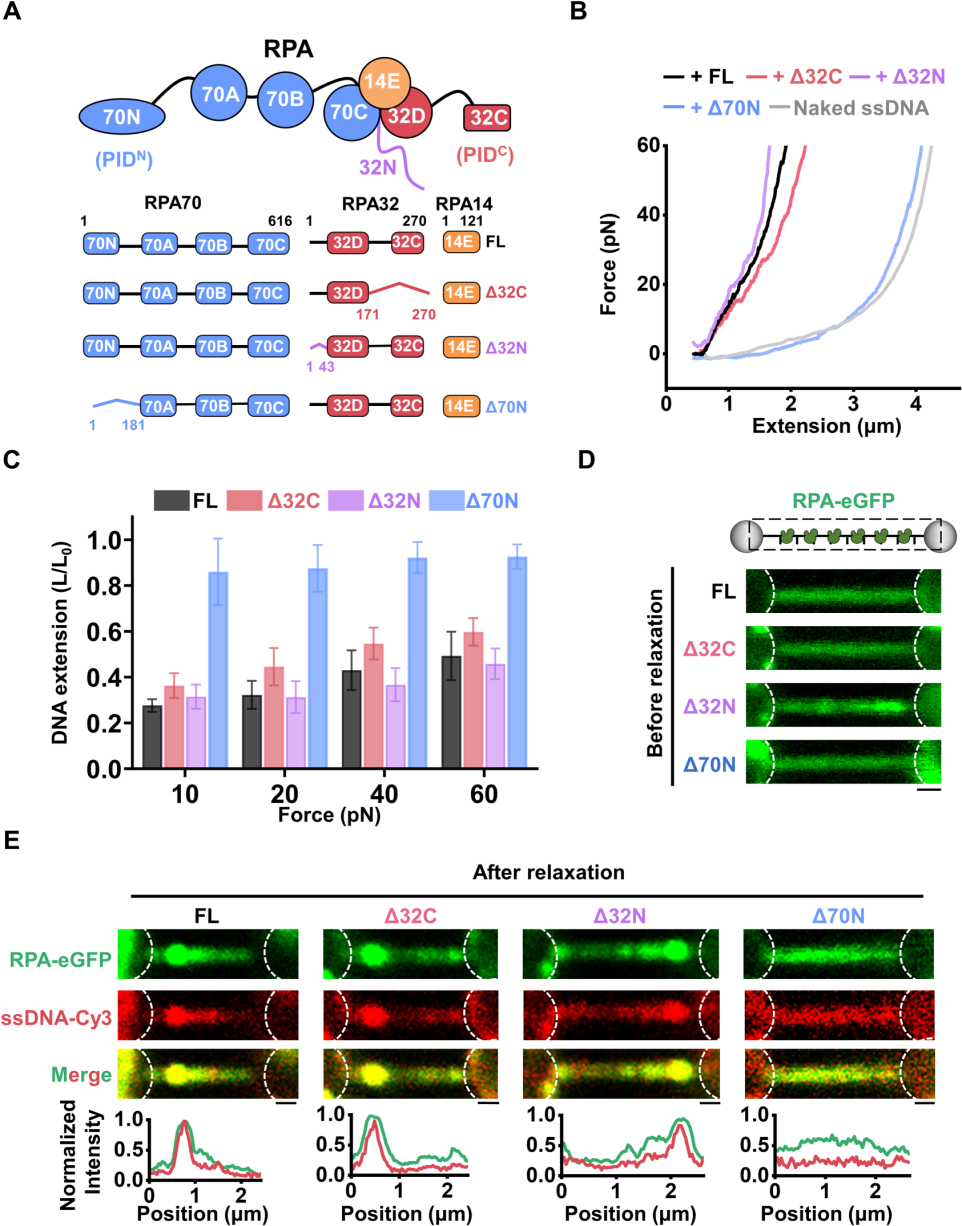
RPA70N-mediated ssDNA condensation. (**A**) Schematic of the three domains of RPA-FL and its three truncated variants. RPA70, RPA32, and RPA14 are colored blue, red, and yellow, respectively. (**B**) Representative force–extension curves of ssDNA bound by RPA-FL and the three variants (40 nM) with a relaxation time of 30 s. The gray line represents the force–extension curve of naked ssDNA. (**C**) The normalized DNA extension (*L/L*_0_) under different forces obtained from force–extension curves of RPA-bound ssDNA after 30 s of relaxation in the presence of 40 nM RPA-FL (*n* = 6), RPA-Δ32C (*n* = 6), RPA-Δ32N (*n* = 5), and RPA-Δ70N (*n* = 8), respectively. *L*_0_ represents the length of naked ssDNA under the indicated force. The data are presented as mean ± SD. (**D**) Representative kymographs showing RPA-FL and its variants fused with eGFP binding to dUTP-Cy3 labeled ssDNA (7.4 knt) before relaxation. Scale bar, 0.5 μm. (**E**) Representative kymographs showing RPA-FL and its variants fused with eGFP binding to dUTP-Cy3 labeled ssDNA (7.4 knt) after relaxation. The corresponding fluorescence intensity profiles of the examined molecules are displayed below the kymographs. Scale bar, 0.5 μm.

Next, we investigated the respective roles of RPA domains in RPA-induced ssDNA condensation. The requirement of multiple RPAs for ssDNA condensation suggested the potential involvement of protein–protein interactions (PPIs) in this process. RPA70N and RPA32C are the two protein-interacting domains within the heterotrimeric complex of RPA [20, 38, 61]. Additionally, RPA32N is acknowledged to contain an IDR that may also participate in PPIs [50]. Thus, we constructed three truncated RPA variants by truncating RPA32C, RPA32N, and RPA70N (termed RPA-Δ32C, RPA-Δ32N, and RPA-Δ70N), respectively (Fig. 3A). By stretching the ssDNA coated by these variants, we found that only the depletion of RPA70N abolishes ssDNA condensation induced by RPA (Fig. 3B and C). The force–extension curves of ssDNA bound by RPA-Δ32C and RPA-Δ32N reveal ssDNA condensation ratios comparable to those of full-length RPA (RPA-FL) (Fig. 3B and C). Consistent with these mechanical measurements, fluorescence imaging analysis revealed that ssDNA molecules bound by RPA-Δ32C and RPA-Δ32N still exhibited localized bright foci, whereas ssDNA bound by the RPA-Δ70N variant displayed a comparatively uniform fluorescence signal (Supplementary Fig. S5). These data highlight the essential role of RPA70N in ssDNA condensation.

To further address how RPA70N mediates ssDNA condensation, we labeled the RPA-FL and the three variants with eGFP (RPA-FL-eGFP, RPA-Δ32C-eGFP, RPA-Δ32N-eGFP, and RPA-Δ70N-eGFP) and visualized their status on ssDNA. Electrophoretic mobility shift assays validated that the eGFP fusion does not impair the DNA-binding activity of RPA (Supplementary Fig. S1B). As reflected by the comparable fluorescence intensities in the single-molecule assay, all four proteins bound to ssDNA efficiently (Fig. 3D and Supplementary Fig. S6). In contrast, upon relaxation and stretching, RPA-FL-eGFP was monitored to coalesce into condensates on the ssDNA, and these condensates largely overlap with the condensed ssDNA-Cy3 signal (Fig. 3E). Similarly, RPA-Δ32C-eGFP and RPA-Δ32N-eGFP proteins also exhibited a coalescence behavior along the ssDNA (Fig. 3E). Nevertheless, unlike these three RPA proteins, RPA-Δ70N-eGFP maintained a uniform binding pattern on the ssDNA after the relaxation rather than undergoing protein coalescence (Fig. 3E and Supplementary Fig. S7). Taken together, ssDNA-bound RPA, through the RPA70N domain, undergoes multimerization along ssDNA, and this process causes ssDNA co-condensation.

### RPA, via RPA70N, undergoes phase separation to condense ssDNA

**Figure 4.**
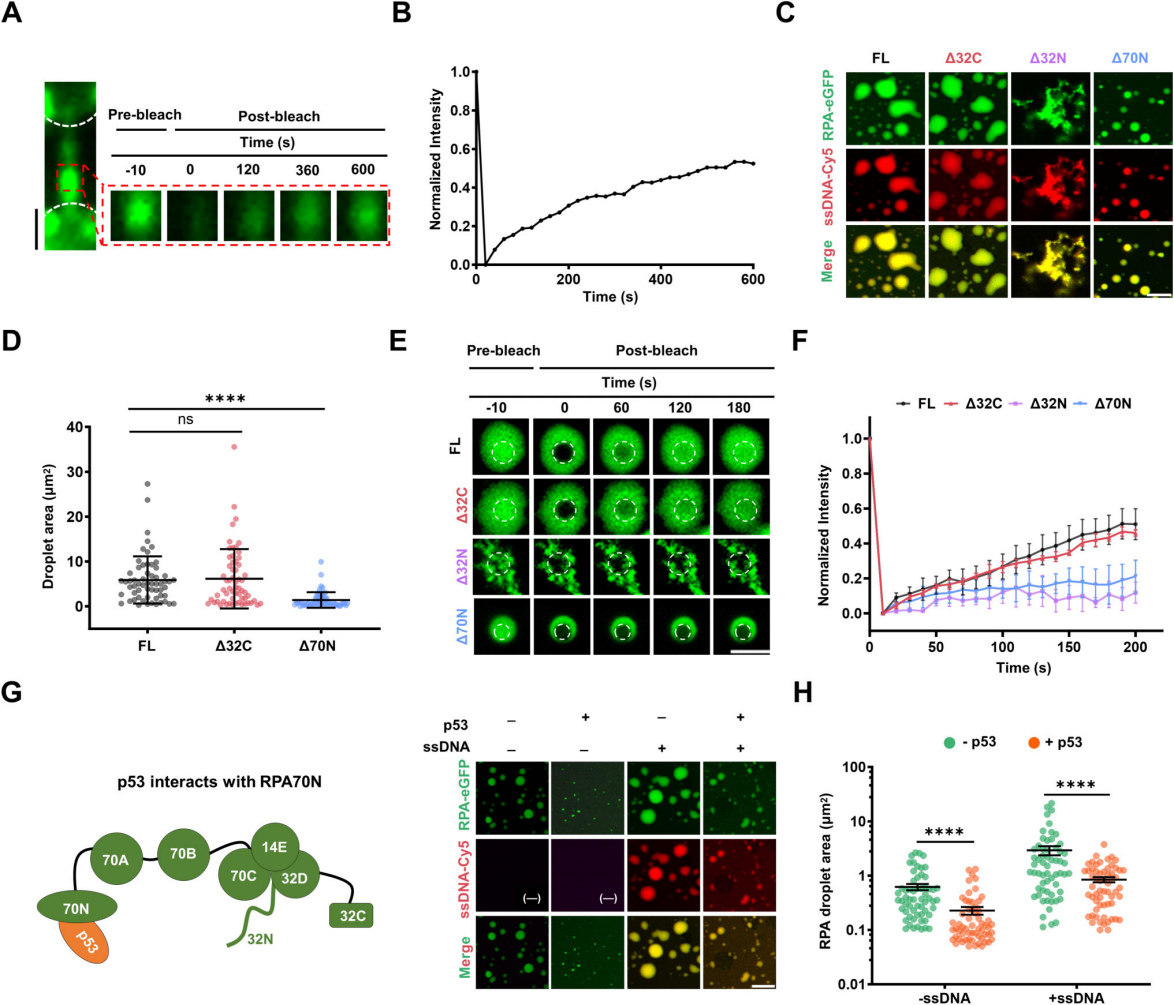
RPA–ssDNA co-phase separation leads to the condensation. (**A**) The fluorescence images of a single DNA-bound RPA-eGFP condensate at different time points before and after the photobleaching in the protein channel. (**B**) The quantified fluorescence normalized intensity at various time points before and after the photobleaching. Normalized fluorescence intensity extracted from raw images is shown as a function of time. (**C**) Representative fluorescence images showing phase separation of 10 μM RPA-FL, RPA-Δ32C, RPA-Δ32N, and RPA-Δ70N in the presence of 1.7 μM 33-nt ssDNA-Cy5. (**D**) Statistical analysis of condensate sizes for RPA-FL and its variants obtained from (**C**). Data are presented as mean ± SD, with *n* = 60 condensates from three independent experiments. Statistical significance is determined using the Kruskal–Wallis test for multiple comparisons (*P* > .05, n.s., ****P < .0001). (**E**) Representative fluorescence images illustrating photobleaching and subsequent fluorescence recovery of RPA-FL and its variants. Scale bar, 2 μm. (**F**) Quantification of FRAP data for RPA-FL and its variants. The data represent the mean ± SD from three independent experiments. (**G**) Representative fluorescence images of RPA-eGFP droplets in the presence and absence of 10 μM p53 and 1.7 μM 33-nt ssDNA-Cy5, respectively. Scale bar, 5 μm. (**H**) Quantification of RPA condensate sizes. Data are presented as mean ± SEM, with *n* = 60 condensates from three independent experiments. ****P < .0001. Mann–Whitney test.

Next, we sought to identify the driving force behind the RPA-mediated ssDNA condensation. Given the phase separation capability of RPA [50], it may co-phase separate with coated ssDNA to form co-condensates, as observed in the OT assays. To test this hypothesis, we first conducted the FRAP assay on RPA-eGFP condensates bound to ssDNA and observed fluorescence intensity recovery within a few minutes (Fig. 4A and B). Thus, it is highly likely that ssDNA-bound RPA-eGFP condensates exist in a phase-separated state.

To systematically characterize the co-phase separation ability of RPA with ssDNA, we examined the status of RPA-FL-eGFP and the three truncated variants in the presence of a 33-nt Cy5-labeled ssDNA. As shown in Fig. 4C, RPA-FL-eGFP underwent phase separation to form spherical co-condensates with the ssDNA. Moreover, either increasing the NaCl concentration or incorporating 1,6-hexanediol attenuated RPA phase separation ability (Supplementary Fig. S8A and B). Thus, RPA employs both electrostatic and hydrophobic interactions for phase separation. RPA-Δ32C-eGFP exhibited a similar phase-separation behavior, with a recovery trend after photobleaching comparable to RPA-FL-eGFP (Fig. 4C–F). However, as manifested by the reduced sizes of the RPA-Δ70N-eGFP–ssDNA co-condensates and the higher saturation concentration, the depletion of RPA70N significantly attenuated RPA phase separation ability (Fig. 4C and D; Supplementary Fig. S8C and D). Additionally, the co-condensates scarcely recovered the fluorescence signals after photobleaching, suggesting their poor fluidity (Fig. 4E and F). On the other hand, unlike the other three RPA proteins, RPA-Δ32N-eGFP was found to aggregate with ssDNA into irregular-shaped condensates with much lower fluorescence recovery from FRAP (Fig. 4C–F). Control experiments using unlabeled RPAs confirmed their inherent phase separation or aggregation activities (Fig. 4C and Supplementary Fig. S8E). Therefore, RPA70N and RPA32N are two key domains responsible for RPA phase separation. The inability of RPA-Δ70N-eGFP to condense ssDNA and its impaired phase separation further support a phase separation–driven mechanism of RPA-mediated ssDNA condensation.

Considering the indispensable role of RPA70N in RPA phase separation, it is plausible that the sequestration of RPA70N could also act as a means to affect its phase separation ability. To test that, we performed the *in vitro* RPA phase separation assays in the presence of p53, a crucial regulator in HR that directly binds to RPA via RPA70N (Fig. 4G) [49]. As expected, the presence of p53 diminished the size of the RPA condensates both in the presence and absence of ssDNA (Fig. 4G and H). In contrast, the size of the RPA-Δ70N-eGFP condensate was unaffected in the presence of p53, implying that p53 binding to the RPA70N domain impairs its phase separation (Supplementary Fig. S9A and B). Moreover, the OT assays revealed an inverse correlation between the RPA–ssDNA co-condensate size and the p53 concentration (Supplementary Fig. S9C). Control experiments ruled out the possibility that these observations are due to the impaired ssDNA-binding ability of RPA by p53 (Supplementary Fig. S10A). Based on the force–extension relationship of the RPA-coated ssDNA, increasing p53 concentration significantly counteracts the extent of ssDNA condensation induced by RPA (Supplementary Fig. S9D and E). Notably, at a 1:1 stoichiometric ratio, p53 can destabilize the condensed RPA–ssDNA nucleoprotein complex, though this regulatory effect is not evident on pre-formed RPA condensates (Supplementary Fig. S10B).

Our findings demonstrate that RPA, via RPA70N and RPA32N, co-phase separates with ssDNA. Irregular morphologies of RPA-Δ32N condensates manifest the dynamical arrest state of the nucleoprotein complex, whereas the smaller RPA-Δ70N condensates reflect the importance of RPA70N in RPA multimerization.

### DNA damage triggers RPA phase separation *in vivo*

**Figure 5.**
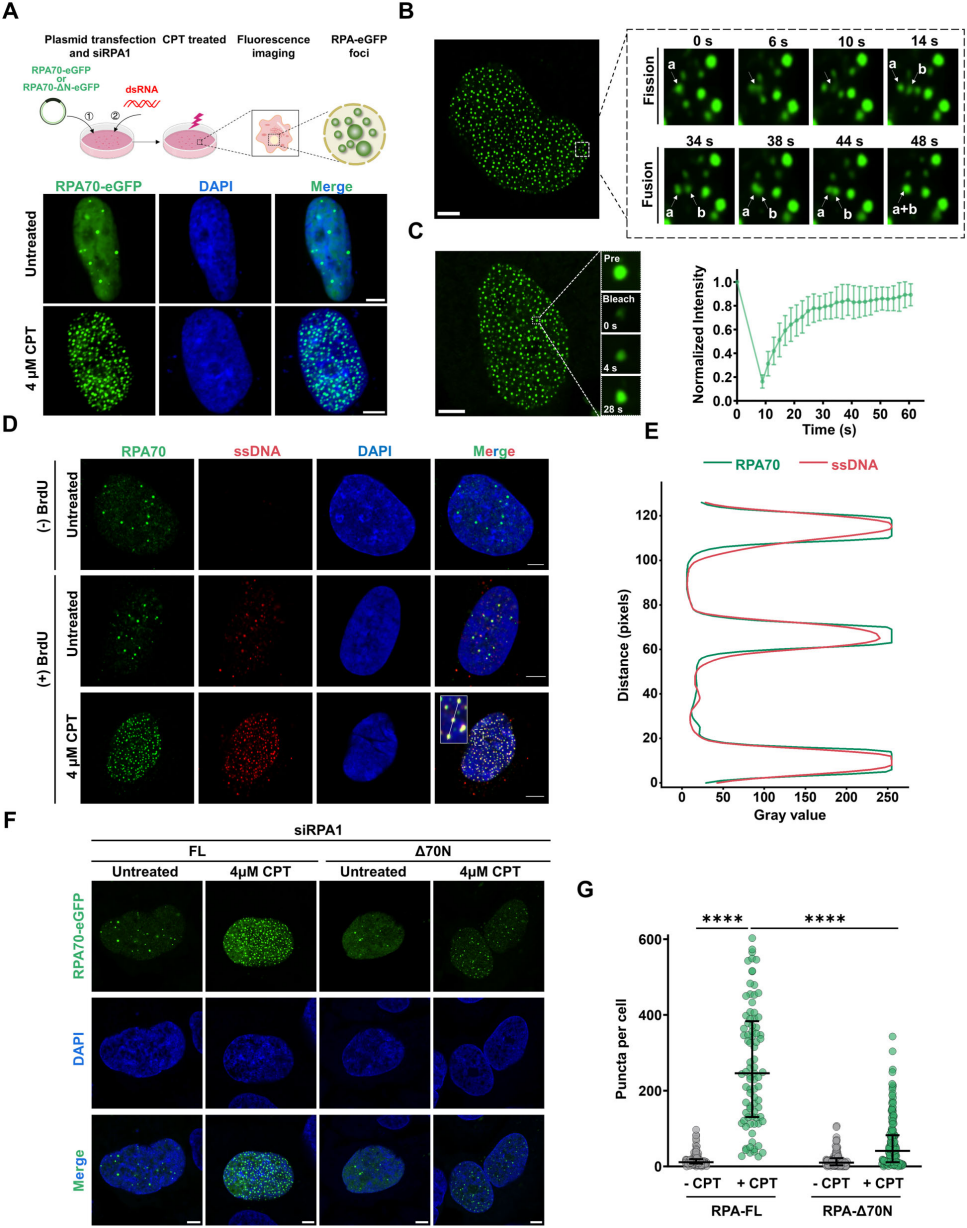
RPA70N-mediated RPA–ssDNA co-phase separation *in vivo*. (**A**) Schematic representation of the *in vivo* experimental workflow (top). Exogenous expression of RPA70-eGFP or RPA-Δ70N-eGFP was performed prior to the knockdown of endogenous RPA1. Live-cell imaging of RPA-eGFP foci in U2OS cells treated with DMSO or 4 μM CPT (bottom). Scale bar, 5 μm. (**B**) Representative fluorescence images showing the fission and fusion of RPA-eGFP foci in CPT-treated U2OS cells. Scale bar, 5 μm. (**C**) Representative images of the FRAP experiment with RPA70-eGFP foci in CPT-treated U2OS cells (left). Scale bar, 5 μM. Normalized fluorescence recovery is shown on the right. Data are presented as mean ± SD, with *n* = 32. (**D**) A representative immunofluorescence image of spermatocyte chromosomal spreads stained for RPA70 (green), ssDNA (red), and DAPI (blue). (**E**) Intensity profiles of RPA 70 and ssDNA signals in the cell (white line in panel D). (**F**) Representative images of RPA-eGFP and RPA-Δ70N-eGFP foci formation in U2OS cells treated with DMSO or CPT. Scale bar, 5 μm. (**G**) Quantification of image data for RPA-eGFP and RPA-Δ70N-eGFP foci per cell. Data are presented as median ± IQR, with *n* = 110, 85, 109, and 152 cells (from left to right). *P* > .05 (n.s.), ****P < .0001. Mann–Whitney test.

Given that RPA can form co-condensates with ssDNA *in vitro*, we next asked whether RPA–ssDNA condensates exist as an intermediate in HR-directed DSB repair. We transfected RPA-eGFP into U2OS cells to address it and observed a foci staining pattern within the nuclei (Fig. 5A). Subsequently, we used CPT to induce DSBs in the cells and monitored the behavior of RPA-eGFP during the repair process [62, 63]. Remarkably, the CTP-treated cells showed a significant increase in the number of the RPA-eGFP foci (Fig. 5A). Similar observations were also made with endogenous RPA upon CPT treatment, suggesting an inherent characteristic of RPA multimerization (Supplementary Fig. S11). High-magnification imaging revealed that RPA-eGFP formed spherical droplets characterized by high mobility and frequent fusion and fission (Fig. 5B). In addition, FRAP analysis of CPT-induced RPA foci revealed a rapid fluorescence signal recovery within 60 s after photobleaching (Fig. 5C). These findings support the occurrence of RPA phase separation during DSB repair and are consistent with our *in vitro* results.

To provide direct evidence that RPA undergoes co-phase separation with ssDNA *in vivo*, we labeled ssDNA with BrdU [64, 65] and induced DSBs using CPT. By performing co-staining with RPA and BrdU antibody, we found that CPT treatment significantly increased ssDNA foci formation and RPA extensively colocalized with these ssDNA foci (Fig. 5D and E), indicating that RPA forms co-condensates with ssDNA at DSB sites. To further validate this, we examined the *in vivo* phase separation ability of RPA-Δ70N, which was expected to be attenuated due to the depletion of RPA70N. To mitigate the influence of endogenous RPA, we knocked down endogenous *RPA1* in U2OS cells and transfected them with RPA70-eGFP and RPA70-ΔN-eGFP, respectively (Fig. 5F and Supplementary Fig. S12). Following CPT treatment, the number of RPA70-Δ70N-eGFP foci was significantly reduced compared with RPA70-eGFP (Fig. 5G). Therefore, RPA indeed undergoes phase separation in the process of DSB repair, which is possibly coupled with ssDNA condensation.

### RPA–ssDNA co-condensates facilitate the local enrichment of RAD51

**Figure 6.**
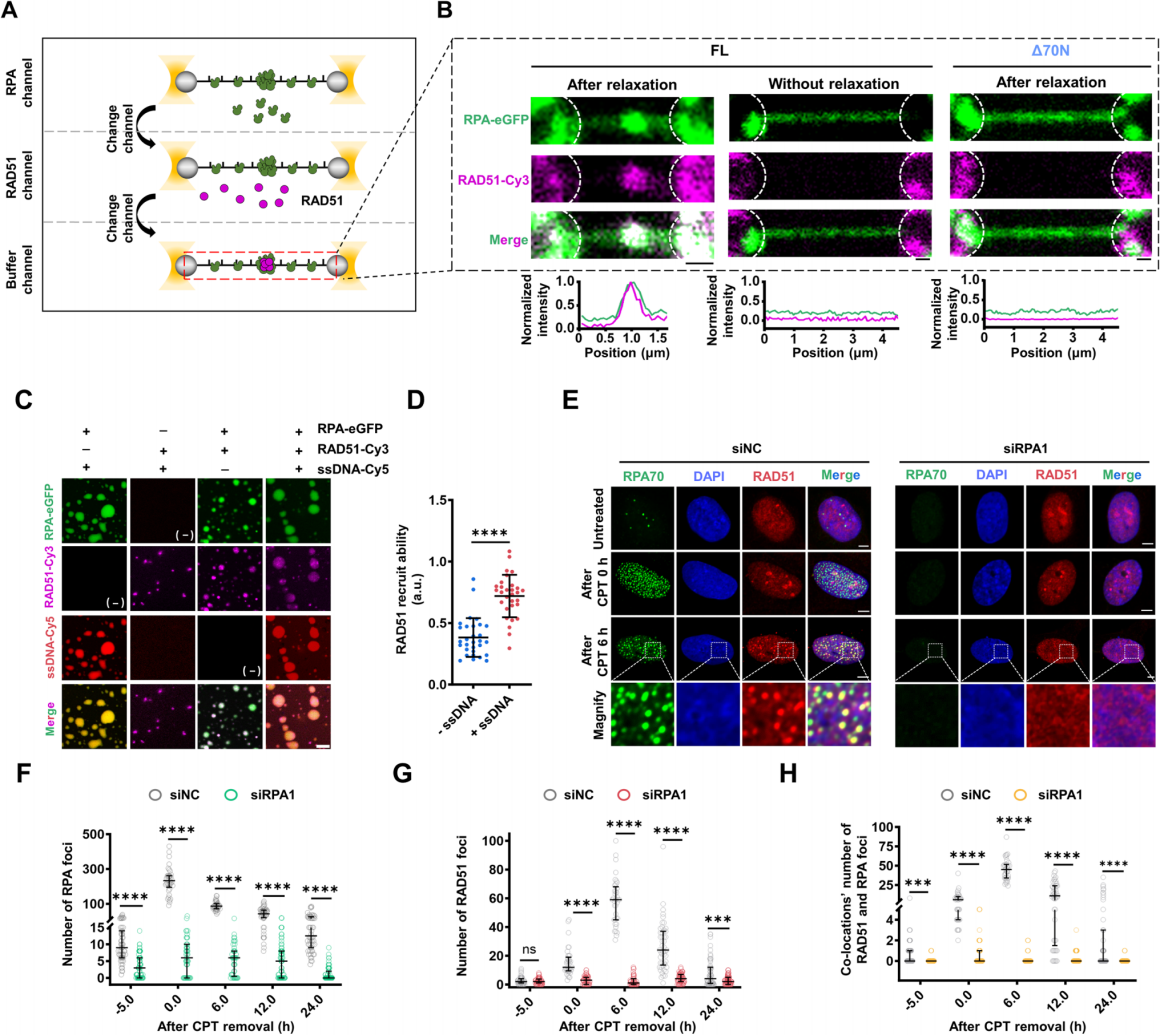
RPA–ssDNA co-condensates promote RAD51 recruitment. (**A**) Schematic representation of the experimental procedures. RPA-eGFP-bound ssDNA is relaxed and stretched to form RPA–ssDNA co-condensates. Subsequently, the RPA-bound ssDNA with the condensate is transferred to the RAD51-Cy3 channel. (**B**) Representative kymographs show RAD51-Cy3 (1 μM) binding to RPA-FL-eGFP or RPA-Δ70N-eGFP-bound ssDNA (12.3 knt) with or without relaxation. The corresponding fluorescence intensity profiles of the examined molecules are displayed below the kymographs. (**C**) Representative fluorescence images of 10 μM RPA-eGFP droplets in the presence and absence of 1.7 μM 33-nt ssDNA-Cy5 and 5 μM RAD51-Cy3. Scale bar, 5 μm. (**D**) The ability of RPA to recruit RAD51 is quantified using the ratio of fluorescence intensity of RPA-eGFP to that of RAD51-Cy3 in each droplet. The data are shown as mean ± SD, with *n* = 60 from three independent experiments. ****P < .0001. Mann–Whitney test. (**E**) Representative images of RPA-eGFP and RAD51 foci in U2OS cells treated with either DMSO or 4 μM CPT after knockdown of endogenous RPA. (**F**) Quantification of RPA-eGFP foci per cell in U2OS cells treated by CPT for -5 h (siNC, *n* = 55; siRPA70, *n* = 60), 0 h (siNC, *n* = 37; siRPA70, *n* = 45), 6 h (siNC, *n* = 37; siRPA70, *n* = 45), 12 h (siNC, *n* = 49; siRPA70, *n* = 60), and 24 h (siNC, *n* = 58; siRPA70, *n* = 61). Data are presented as median ± IQR, *P* > .05 (n.s.), ****P < .0001. Mann–Whitney test. (**G**) Quantification of RAD51 foci per cell in U2OS cells treated by CPT for -5 h (siNC, *n* = 55; siRPA70, *n* = 60), 0 h (siNC, *n* = 37; siRPA70, *n* = 45), 6 h (siNC, *n* = 37; siRPA70, *n* = 45), 12 h (siNC, *n* = 49; siRPA70, *n* = 60), and 24 h (siNC, *n* = 58; siRPA70, *n* = 61). Data are presented as median ± IQR, *P* > .05 (n.s.), ****P < .0001. Mann–Whitney test. (**H**) Quantification of RAD51 foci co-localizing with RPA foci per cell in U2OS cells treated by CPT for -5 h (siNC, *n* = 55; siRPA70, *n* = 60), 0 h (siNC, *n* = 37; siRPA70, *n* = 45), 6 h (siNC, *n* = 37; siRPA70, *n* = 45), 12 h (siNC, *n* = 49; siRPA70, *n* = 60), and 24 h (siNC, *n* = 58; siRPA70, *n* = 60). Data are presented as median ± IQR, *P* > .05 (n.s.), ****P < .0001. Mann–Whitney test.

Having demonstrated that RPA exists in a phase-separated state after DNA damage, we next aimed to elucidate its functional role in DSB repair. In HR-directed DSB repair, the recombinase RAD51 is supposed to identify ssDNA-bound RPA and assemble a nucleoprotein filament for homology search [4, 13, 15]. However, RAD51 alone cannot directly load onto the RPA-coated ssDNA [23, 24, 66]. We therefore asked whether RAD51 responds differently to phase-separated RPA–ssDNA co-condensates. To this aim, we prepared Cy3-labeled human RAD51 (RAD51-Cy3) and recorded its fluorescence signal on an RPA-coated ssDNA molecule (Fig. 6A). To our surprise, following a 30-s incubation in a 1 μM RAD51-Cy3 channel, we observed the emergence and progressive accumulation of the RAD51-Cy3 signal on RPA–ssDNA co-condensates. Control experiments excluded the possibility that RAD51 binding arises from exposed ssDNA due to incomplete RPA coating within the condensates (Supplementary Fig. S13). In contrast, no RAD51 signals were detected on the uniformly bound RPA (Fig. 6B and Supplementary Fig. S14). To better mimic physiologically relevant DNA structures generated during end resection, we designed an ss/dsDNA hybrid substrate. Consistent with our observations with the ssDNA substrate, RPA formed condensates on the ssDNA region within the hybrids, which also facilitated the local enrichment of RAD51 (Supplementary Fig. S15).

To further substantiate the role of the RPA–ssDNA condensate, we revisited the OT assay by either preventing ssDNA relaxation or using RPA-Δ70N, both of which were designed to inhibit the formation of the RPA–ssDNA condensate. As expected, RAD51 association with RPA-coated ssDNA was negligible in the two experiments (Fig. 6B). Interestingly, although the depletion of RPA32N impairs RPA phase separation, the resulting RPA complex retains its ability to support RAD51 enrichment (Supplementary Fig. S16). These results suggest that RPA–ssDNA co-condensates provide a favorable platform for RAD51 gathering.

Next, we sought to explore the mechanism underlying RAD51 enrichment. To explore this, we performed the phase separation experiments with the presence and absence of RAD51, RPA, and ssDNA. Indeed, RAD51-Cy3 can undergo co-condensation with RPA-eGFP alone and with the combination of ssDNA-Cy5 and RPA-eGFP (Fig. 6C). Interestingly, the presence of ssDNA significantly enhanced RAD51 partitioning into RPA condensates, as indicated by increased RAD51 fluorescence intensity per unit area (Fig. 6D). In contrast, the absence of RPA significantly impaired the compartmentalization of RAD51 with ssDNA (Fig. 6E).

To provide *in vivo* support, we monitored the localization of RAD51 following the induction of RPA–ssDNA condensates in U2OS cells. Upon CPT treatment, RPA–ssDNA condensates were first detected in the cells, whereas RAD51 exhibited only a few large foci structures (Fig. 6E). Following the removal of CPT, which facilitates the DSB repair process in cells, co-localization of the RAD51 and RPA foci signals was immediately noticed (Fig. 6E). Given the resolution limits of fluorescence microscopy, we interpret this as co-localization rather than definitive evidence of co-condensation. It is noteworthy that during meiotic DSB repair, RPA is also recruited to meiotic DSB sites and colocalizes with RAD51 (Supplementary Fig. S17) [67]. Statistical analysis revealed a reduction in the number of the RPA foci after the CPT removal, while the number of the RAD51 foci decreased after an initial increase in U2OS cells (Fig. 6F and G). Notably, more than half of the RAD51 foci were spatially co-localized with the RPA foci at different time points (Fig. 6H). The gradual decreases in RAD51 and RPA foci were expected as the DSBs were successfully repaired with time (Supplementary Fig. S18). In addition, RAD51 foci were significantly reduced upon dissolution of RPA condensates following RPA70 knockdown in U2OS cells (Fig. 6E), supporting a role for RPA assemblies in facilitating RAD51 gathering in cells.

To directly assess the impact of RPA-Δ70N on repair efficiency, we tracked DSB repair kinetics by monitoring a well-established marker γH2A.X for DSBs in eukaryotes [68]. Following CPT treatment in RPA1-knockdown cells, those expressing exogenous RPA70-eGFP exhibited pan-nuclear γH2A.X staining, which progressively diminished over time and returned to pre-damage levels within 24 h, indicating successful repair. In stark contrast, RPA1 knockdown cells expressing RPA70-ΔN-eGFP showed a persistent γH2A.X signal that failed to resolve, demonstrating defective DSB repair (Supplementary Fig. S19).

Taken together, our results support a model in which RPA–ssDNA condensates act as hubs that facilitate the local enrichment of RAD51 during HR. These findings highlight a role for phase-separated RPA assemblies in organizing repair factor dynamics during DNA damage response.

## Discussion

In this work, we first developed a streamlined approach for ssDNA generation in the OT-based single-molecule assays. We took advantage of the specialized nuclease activity of RNase H toward RDHs, allowing for the efficient digestion of the ssRNA from paired ssDNA under a low force (Fig. 1). In comparison, our approach is highly effective, avoiding high tension on the DNA template and the involvement of additional DNA structures in the resulting ssDNA [52, 57–59]. In addition to the length and force measured by OT, the simultaneous monitoring of ssDNA status is achieved by combining with confocal fluorescence microscopy, thus offering additional perspectives on the ssDNA measurements.

RPA is a major ssDNA-binding protein in mammalian cells that prevents the coated ssDNA from secondary structure formation and unwanted nucleolytic cleavage and degradation [16, 19, 27, 69]. We investigated the dynamic ssDNA–RPA interactions at the single-molecule level using the newly developed approach. In addition to the dynamic binding to ssDNA, RPA was found to exhibit dynamic PPIs that form phase-separated condensates on ssDNA under appropriate conditions, consistent with a previous study [50]. Meanwhile, the phase separation of RPA gives rise to the simultaneous condensation of ssDNA, resulting in the formation of the RPA–ssDNA co-condensate (Fig. 3E). Surprisingly, this nucleoprotein condensate maintains a stable structure capable of withstanding forces of several hundreds of pN (Supplementary Fig. S4). It is imaginable that the compressed ssDNA is well protected within these condensates. We are unable to completely exclude the possibility that regional local wire coiling of RPA-bound ssDNA initiates partial ssDNA condensation. Nevertheless, as proved by our phase separation and FRAP assays (Fig. 4), RPA exists in a phase-separated state at the end. This would allow the locally wired ssDNA to be absorbed into the condensate, thus forming the RPA-ssDNA co-condensate. Although RPA exhibits phase-separated behavior both *in vitro* and *in vivo*, the higher FRAP recovery observed in cells likely reflects the more dynamic intracellular environment, where RPA condensates are influenced by factors such as molecular turnover, interaction partners, and continuous remodeling of DNA–protein assemblies (Figs 4F and 5C). These factors are expected to promote more rapid and extensive exchange of RPA molecules, leading to enhanced FRAP recovery *in vivo*. We further identified RPA32N and RPA70N as the key domains driving RPA phase separation (Fig. 4). However, these two domains function differently in mediating RPA phase separation. Consistent with a previous study [50], depletion of RPA32N led to the physical aging of fluid-like RPA condensate, characterized by a dynamically arrested state with the appearance of non-spherical morphologies for condensates. Conversely, RPA70N, a protein-interacting domain, likely mediates physical crosslinks within RPA, creating a network-like organization that determines the size of the condensate. As evidenced by the OT assays, RPA70N, rather than RPA32N, plays an indispensable role in ssDNA condensation (Figs 3 and 4).

**Figure 7.**
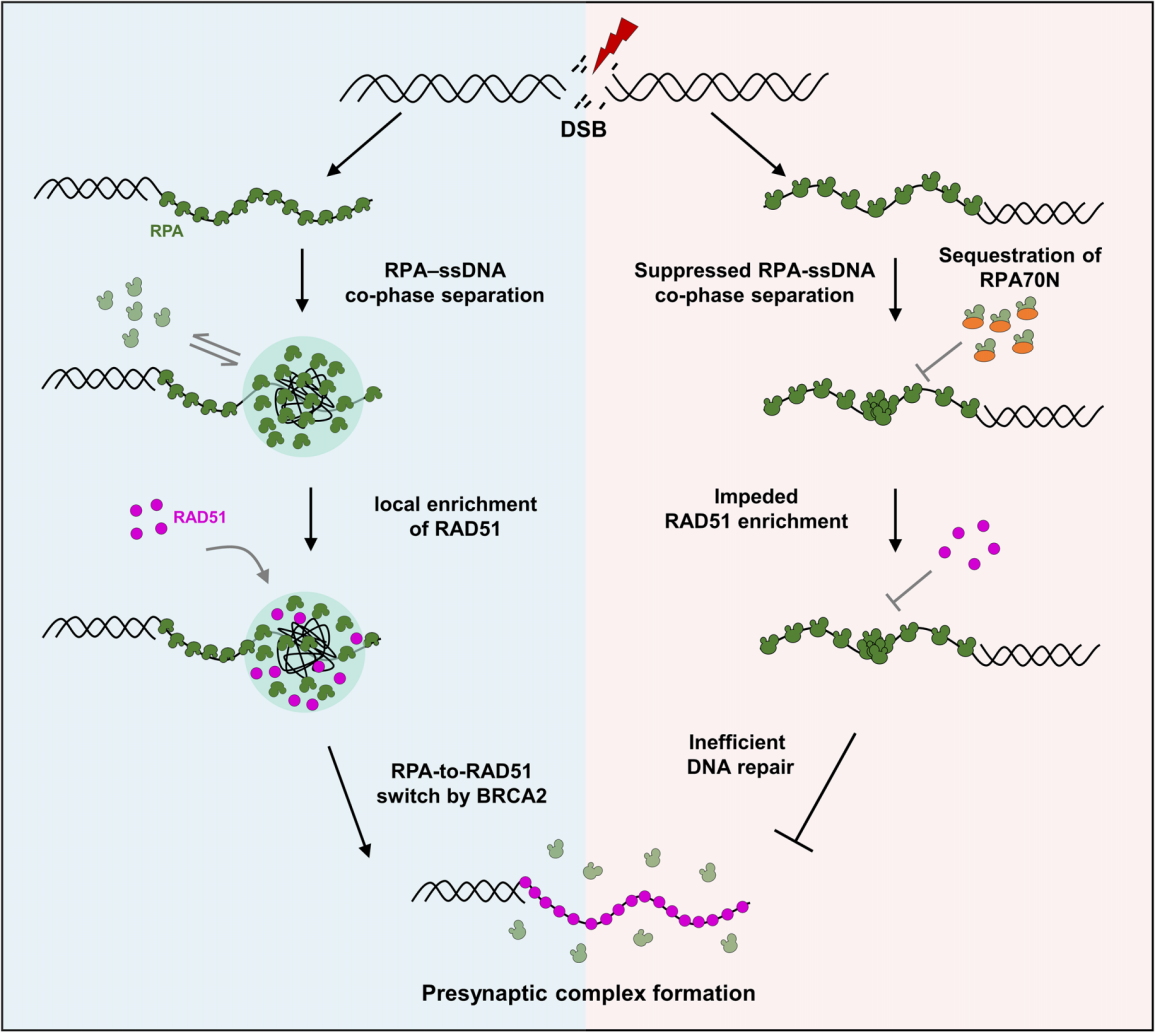
A working model for RAD51 enrichment mediated by RPA–ssDNA co-condensates during HR-directed DSB repair. (Left) In this process, a long 3′ ssDNA overhang generated by helicases and nucleases is rapidly coated by RPA. In the presence of free RPA, ssDNA-bound RPA undergoes phase separation via the RPA70N domain, leading to co-condensation of ssDNA. The resulting RPA–ssDNA co-condensates act as central hubs for the local enrichment of RAD51, preparing for the RPA-to-RAD51 switch by BRCA2. (Right) Disruption of RPA phase separation, e.g. through sequestration of RPA70N by interacting proteins, may reduce RAD51 enrichment and ultimately compromise DSB repair, leading to genomic instability.

Besides ssDNA protection, ssDNA-bound RPA also serves as a platform that coordinates with a set of factors for the downstream processes, particularly the handover of ssDNA to other proteins, such as recombinase and DNA polymerase required in DNA replication, DNA mismatch repair, and HR [27, 70–72]. Our work established the functional role of phase-separated RPA in HR and demonstrated that the RPA–ssDNA co-condensate serves as a gathering center for the downstream protein recruitments. Both *in vitro* and *in vivo* assays revealed that the RPA–ssDNA condensates rather than uniformly coated ssDNA preferentially recruit RAD51 (Fig. 6). Since RPA is involved in multiple DNA metabolic processes, identifying ssDNA-bound RPA that participates in HR is imperative for RAD51. During HR, a long stretch of several kbp of ssDNA is generated that requires a high amount of RPA proteins for the coating [4, 16, 73, 74]. It is conceivable that this requirement and the long stretch of ssDNA facilitate the formation of RPA–ssDNA condensates. In contrast, the ssDNA generated during DNA replication is relatively short (only 150–200 nt) [75–77]. Under physiological conditions, DNA Pol δ synthesizes DNA at a rate of 50 nt/s [78], meaning that RPA remains bound to ssDNA for only ~4 s. This limited binding time on a short ssDNA is possibly insufficient for RPA to accumulate and undergo phase separation, thus avoiding RAD51 recruitment. With these notions in mind, it is tempting to propose a screening mechanism wherein phase-separated RPA condensate formed in HR serves as a marker that specifically signals RAD51 for recruitment (Fig. 7). In support of that, loss of RPA was reported to abrogate recombinase loading to meiotic DSBs completely, and our data also indicated the co-localization of RPA with RAD51 at meiotic DSBs (Supplementary Fig. S17) [67]. The formation of RPA-based biomolecular condensates enriches RAD51 locally. It should be noted that RAD51 enrichment mediated by RPA–ssDNA co-condensates is not simply a consequence of direct RPA–RAD51 interaction, but instead depends on the condensate state. This is supported by the observation that RAD51 is enriched only at RPA–ssDNA co-condensates. This local enrichment may prime ssDNA for the subsequent RPA-to-RAD51 switch and presynaptic filament assembly mediated by the BRCA2–DSS1 complex [4, 23–26].

Our work also hints at a regulatory pathway for recombinase recruitment through RPA70N-interacting proteins, such as p53. In previous studies, p53 was found to intervene in ssDNA protection by sequestering RPA and downregulating HR [48, 49, 79]. Our results showed that p53 does not impair RPA’s ssDNA-binding ability (Supplementary Fig. S10A). Its presence significantly attenuates RPA phase separation (Fig. 4G and H; Supplementary Fig. S9). This attenuation results from the sequestration of RPA70N by p53, which jeopardizes RPA’s PPIs. This regulation is possibly dynamic because phosphorylation of both p53 and RPA was acknowledged to disrupt their interactions [47, 80]. Additionally, p53 oscillations in its nuclear concentration were reported to control droplet formation for enhanced DNA repair [81]. Thus, RPA70N-interacting proteins, like p53, may act as dynamic regulators in HR that control the ssDNA-binding status of RPA, thereby the RAD51 recruitment (Fig. 7). Overall, this work identifies the RPA–ssDNA condensates as an essential intermediate for the recombinase recruitment during HR, deepening our understanding of phase separation in maintaining genome stability.

## Supplementary Material

gkag586_Supplemental_File

## Data Availability

The data underlying this article are available in the article and in its online supplementary material. Additional material or information will be shared on reasonable request to the corresponding author.
