# Supplementary material for: RPA–ssDNA co-phase separation facilitates RAD51 enrichment during homologous recombination
Source: Nucleic Acids Res. 2026 Jun 11;54(11):gkag586. doi: 10.1093/nar/gkag586 (PMC13255724; doi:10.1093/nar/gkag586)
Supplement: gkag586_Supplemental_File [file gkag586_supplemental_file.pdf]

Supplementary Materials for

RPA–ssDNA co-phase separation facilitates RAD51 enrichment during homologous recombination

Yanan Li[1,2,†], Yi Zhao[2,†], Yinghong Chen[3,4,†], Teng Wang[2,5], Lulu Bi[2,6], Yanling Bao[2], Lishuang Chen[2], Xia Zhang[2], Bingkai Cheng[2], Meng Hu[2], Shengli Jing[1], Chao Liu[3,*], Wei Li[3,*], Bo Sun[2,*]

*Correspondence: liuchsdu@163.com (C. Liu), leways@gwcmc.org (W. Li), sunbo@shanghaitech.edu.cn (B. Sun)

This file includes

Supplementary Figures S1 – S19

Supplementary Tables S1 – S2

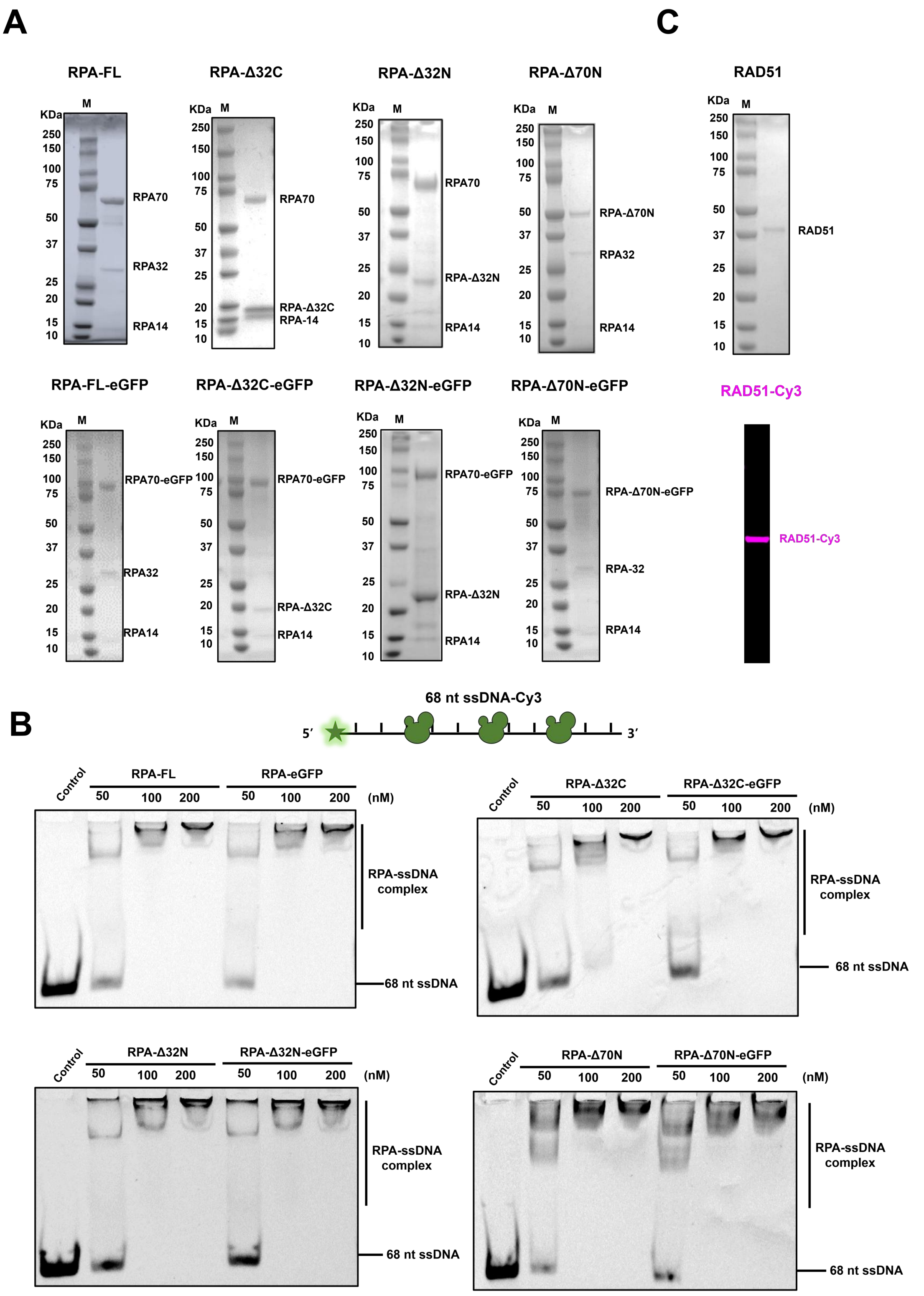

Supplementary Figure S1. Purification of RPA variants and RAD51. **(A)** The representative gels of purified recombinant RPA-FL, RPA-Δ32C, RPA-Δ32N, RPA-Δ70N, RPA-FL-eGFP, RPA-Δ32C-eGFP, RPA-Δ32N-eGFP, and RPA-Δ70N-eGFP proteins. **(B)** Electrophoretic mobility shift assays (EMSA)

comparing the ssDNA-binding abilities of RPA and RPA-eGFP, RPA-∆32C and RPA-∆32C-eGFP, RPA-∆32N and RPA-∆32N-eGFP, and RPA-∆70N and RPA-∆70N-eGFP, respectively. **(C)** The representative gels showing the purification and fluorescence labeling of RAD51.

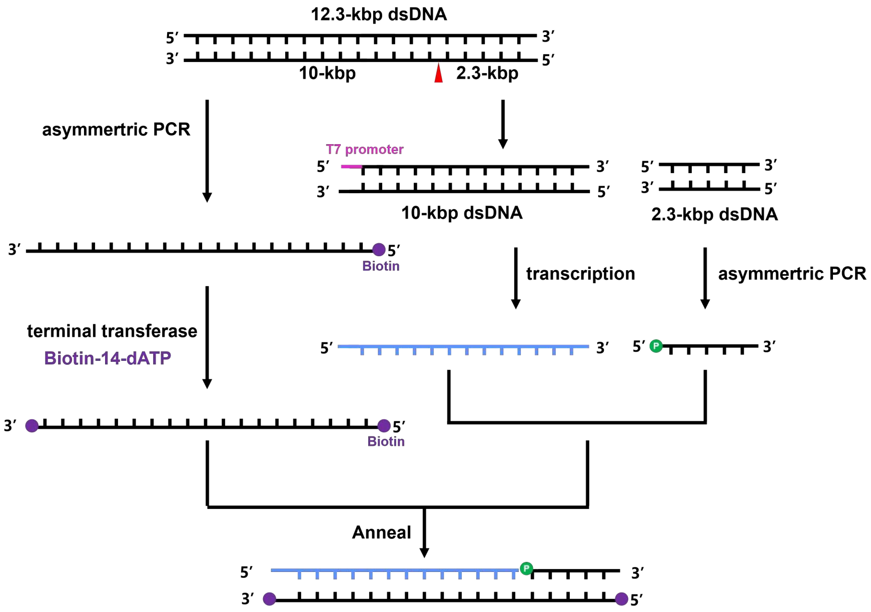

Supplementary Figure S2. Schematic illustration of the construction of the RNA–DNA/dsDNA hybrid template used to generate ss/dsDNA substrates for optical tweezers assays. A 12.3-kbp dsDNA fragment was PCR-amplified from λ DNA. A 12.3-knt ssDNA was generated by asymmetric PCR using a biotin-labeled primer, followed by 3′-end biotinylation with biotin-dATP (APExBIO) and terminal transferase. The 12.3-kbp dsDNA was further divided into 10-kbp and 2.3-kbp fragments. A 10-knt ssRNA was transcribed in vitro from the dsDNA fragment containing a T7 promoter, and a 2.3-knt ssDNA was generated by asymmetric PCR. The final 12.3-kbp RNA–DNA/dsDNA hybrid substrate was assembled by annealing the 12.3-knt ssDNA with the complementary 10-knt ssRNA and 2.3-knt ssDNA fragments.

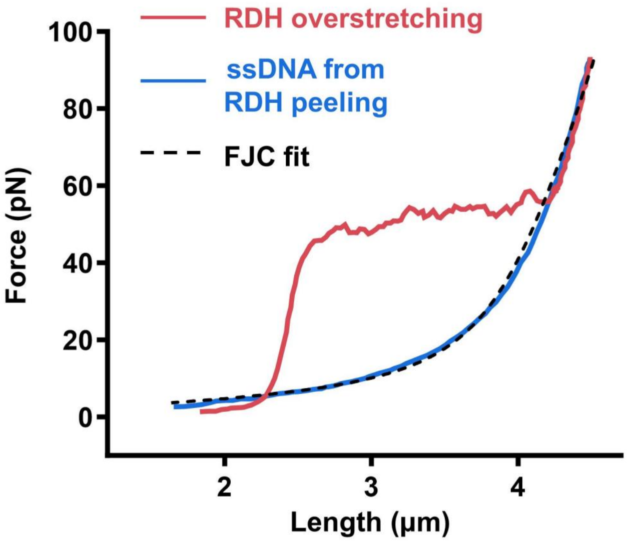

Supplementary Figure S3. A representative force–extension curve of a 7.4-knt ssDNA molecule obtained from the high force-induced RDH peeling. The 7.4-kbp RDH was overstretched to generate the 7.4-knt ssDNA molecule (red). The force–extension relationship of the resulting ssDNA (blue) was fitted with an FJC model (black). The fitting parameters were comparable to the ssDNA from the RNase H-based approach (**Fig. 1E**).

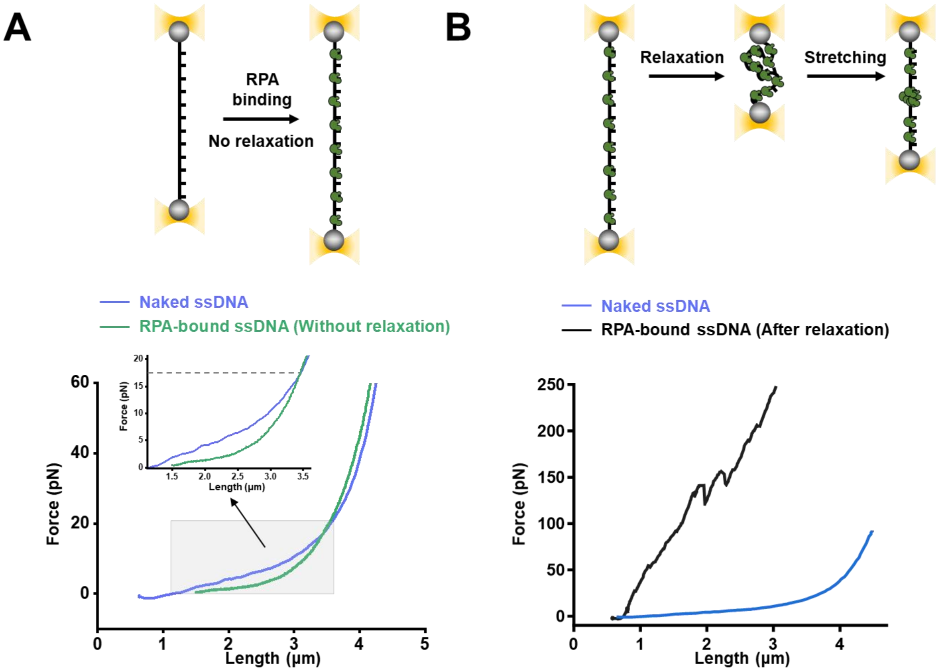

Supplementary Figure S4. Stretching RPA-coated ssDNA with or without relaxation. **(A)** A representative force–extension curve of an RPA-bound ssDNA molecule showing that without relaxation, the contour length of the ssDNA was extended under applied forces of less than approximately 15 pN. **(B)** A representative force–extension curve of an RPA-bound ssDNA molecule showing that after relaxation, the RPA–ssDNA nucleoprotein complex exhibited a decrease in the contour length of ssDNA. Remarkably, the complex can sustain a high disruptive force of 250 pN.

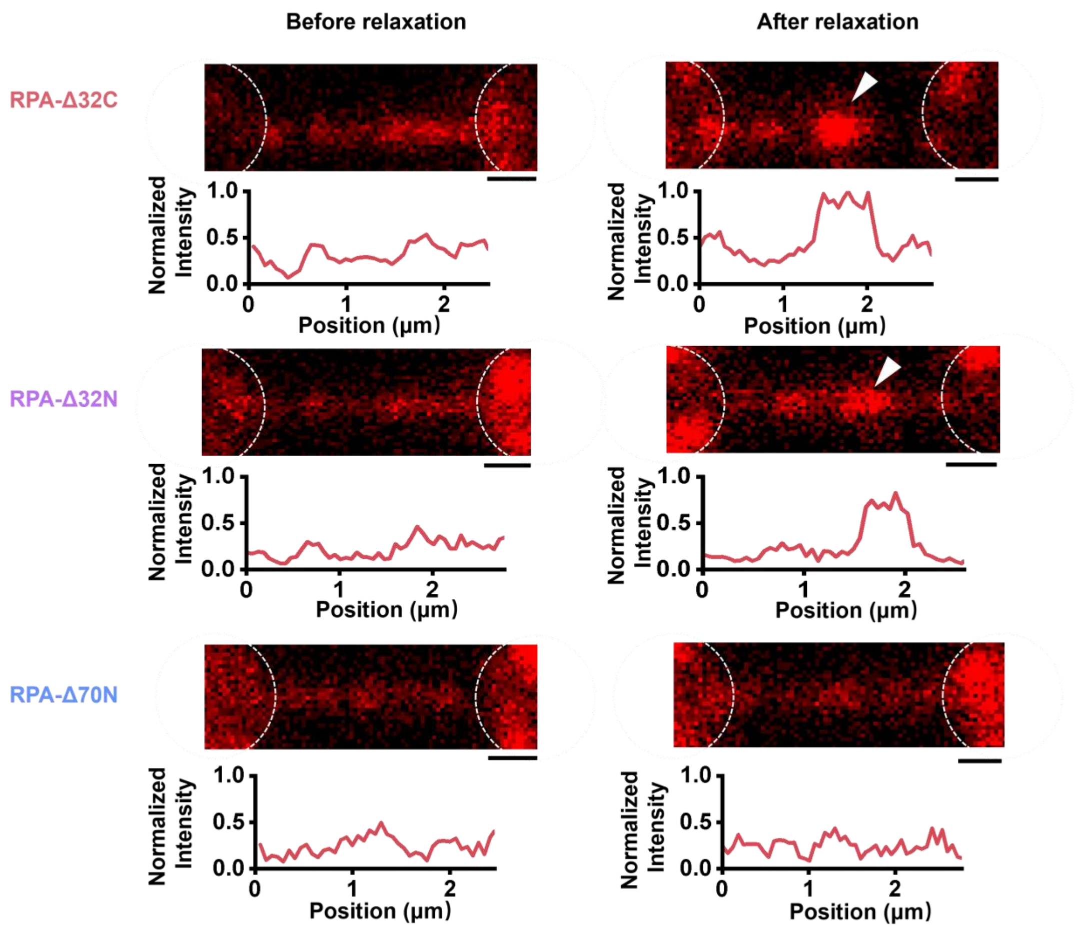

Supplementary Figure S5. A dUTP-Cy3 labeled RPA-bound ssDNA template (7.4 knt) is suspended between two beads manipulated by two optical traps, with confocal lasers scanning before (left) and after (right) the relaxation and stretching processes, respectively. The corresponding fluorescence profiles of the examined ssDNA are shown below the kymographs. Arrows highlight ssDNA condensation. Scale bar, 0.5 μm.

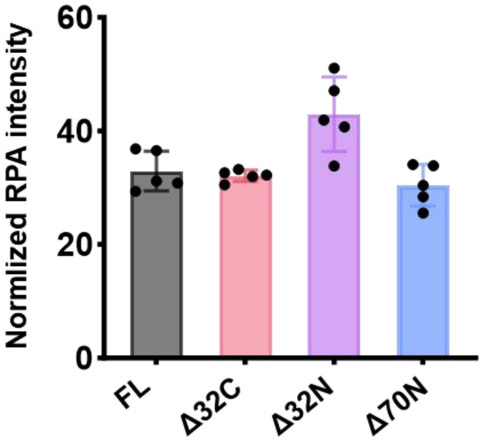

Supplementary Figure S6. Comparison of the fluorescence intensities of ssDNA-binding RPA and variants. The fluorescence intensities of RPA-eGFP, RPA-Δ32C-eGFP, RPA-Δ32N-eGFP, and RPA-Δ70N-eGFP (40 nM) on ssDNA are comparable, suggesting that their ssDNA binding abilities are not impaired after the truncation.

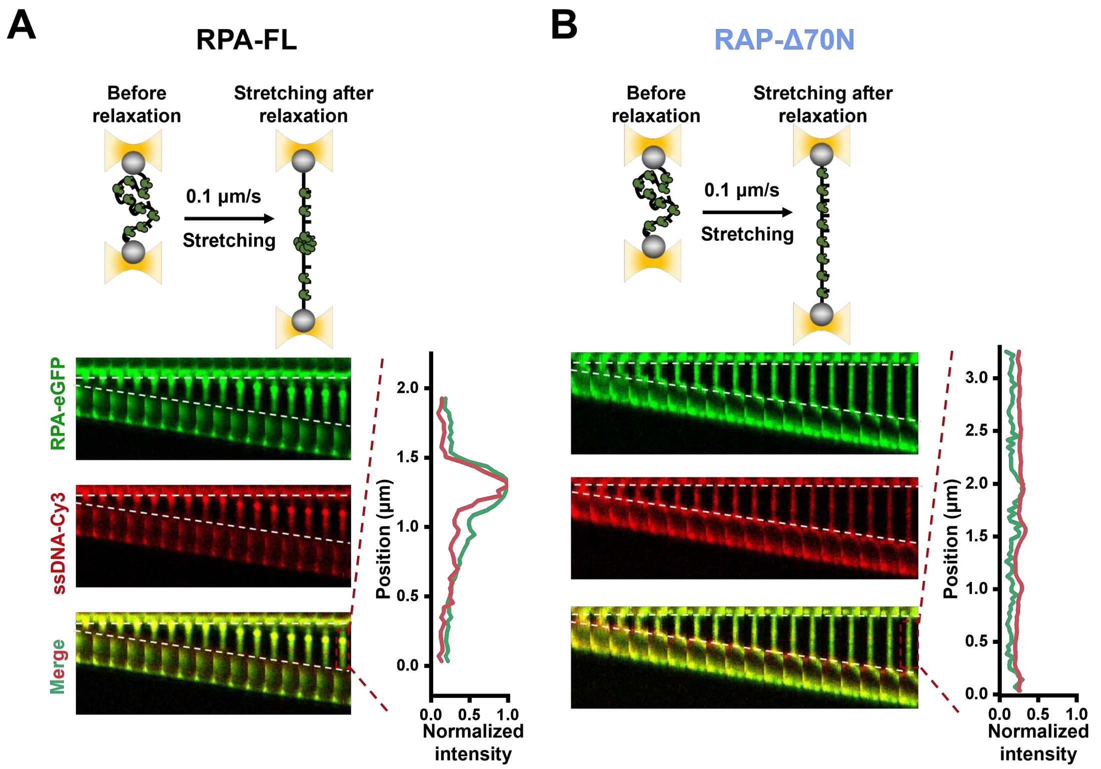

Supplementary Figure S7. Comparison of the PRA-FL- or RPA-Δ70N-bound ssDNA during stretching. (**A-B**) RPA-FL-eGFP- (**A**) or RPA-Δ70N-eGFP-bound (**B**) ssDNA-Cy3 was stretched at a rate of 0.1 μm/s, simultaneously recording the fluorescence signals of both protein (green) and ssDNA (red). For clarity, we scanned a rectangular area in each frame to examine the protein and ssDNA status. The intensity profiles of their last frame showed the regional co-condensation of RPA-FL with ssDNA (**A**) and yet a uniform coating of RPA-Δ70N on ssDNA (**B**).

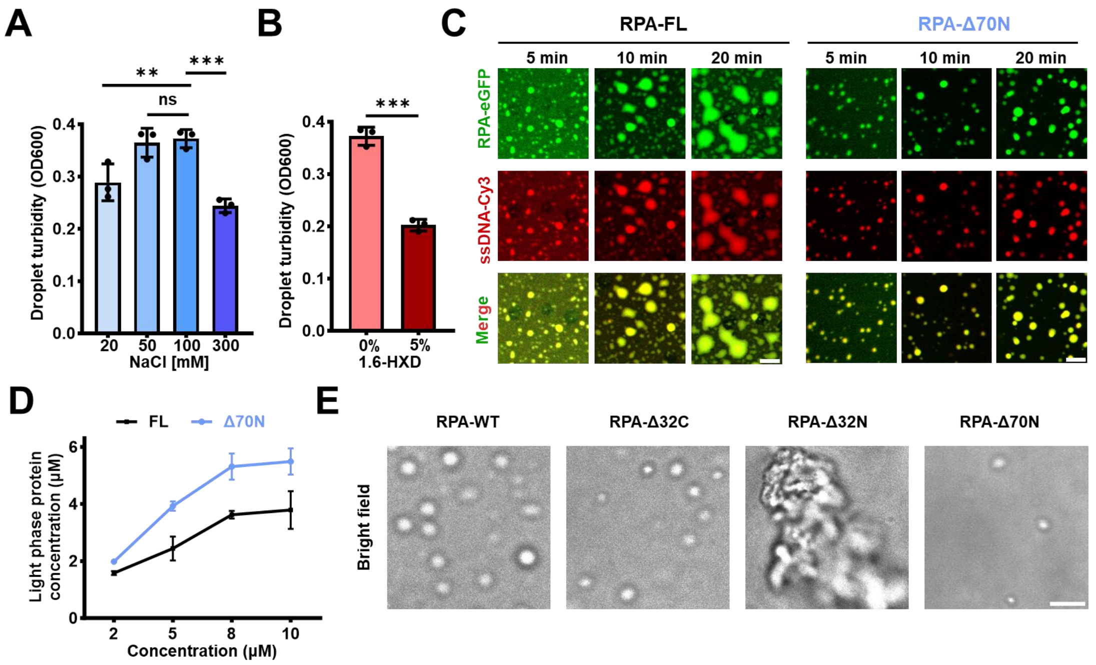

Supplementary Figure S8. Phase separation ability and properties of RPA and its variants**. (A)** Turbidity measurements of 10 μM RPA-eGFP incubated with 1.7 μM ssDNA-Cy5 under varying salt concentration conditions. The data are presented as mean ± SD, with n = 3 from three independent experiments. One-way analysis of variance (ANOVA) with Dunnett's test for multiple comparisons, p > 0.05 (n.s.), **p < 0.01, ***p < 0.001. **(B)** Turbidity measurements of 10 μM RPA-eGFP incubated with 1.7 μM ssDNA-Cy5 in the presence or absence of 5% 1,6-hexanediol. The data are presented as mean ± SD, with n = 3 from three independent experiments. Unpaired t-test, ***p < 0.001. **(C)** The fluorescence images under different incubation periods show that RPA-FL (left) and RPA-Δ70N (right) form co-condensates with ssDNA. However, the average size of the RPA-Δ70N-ssDNA co-condensates is smaller than that of RPA-FL (**Fig. 4C**). Scale bar, 5 μm. **(D)** Saturation concentrations of RPA-FL and RPA-Δ70N. The saturation concentration is the critical protein concentration at which phase separation occurs, with all proteins remaining in the light phase below this threshold. Notably, the protein concentration in the light phase remains at saturation once condensation begins. A centrifugation method was employed to determine the saturation

concentration required for RPA condensation. RPA-FL or RPA-Δ70N was initially prepared at various concentrations in the buffer containing 1.7 μM 33-nt ssDNA-Cy5, 25 mM Tris-HCl (pH 7.5), 100 mM NaCl, 3 mM DTT, 1 mM $MgCl_2$, and 5% (w/v) PEG-8000. After a 20-minute incubation, the RPA condensates were collected by centrifuging at 11,000 rpm for 10 min at 4°C. The concentration of the resulting clarified supernatant, representing the light phase, was then measured using a Colibri spectrophotometer (Berthold). The data are presented as mean ± SEM from two independent experiments. The measured saturation concentrations for RPA-Δ70N are higher than those of RPA-FL, suggesting depletion of RPA70N impairs the RPA phase separation ability. **(E)** The images depict the formation of phase-separated condensates by wild-type RPA and variants (10 μM). Scale bar, 5 μm.

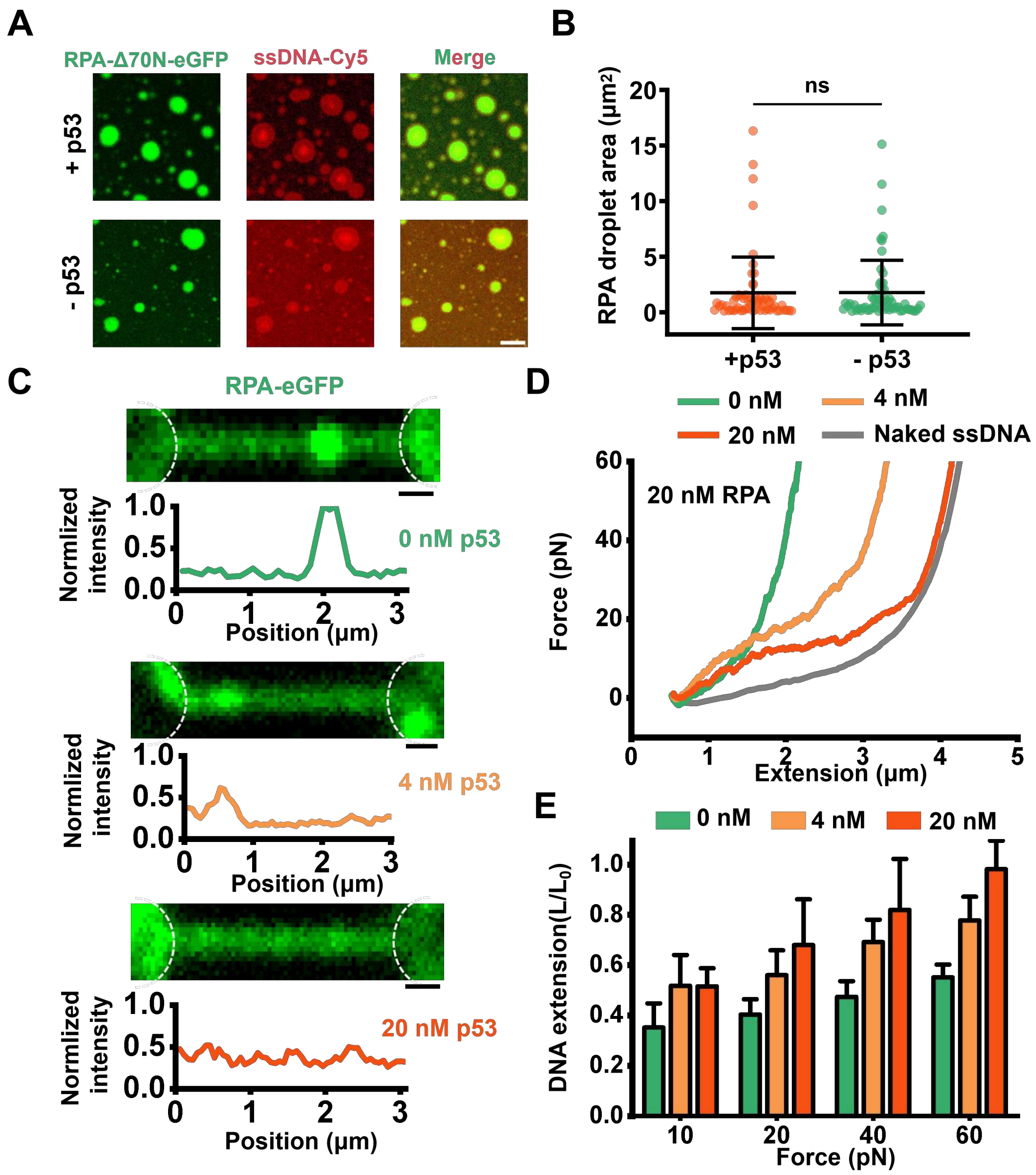

Supplementary Figure S9. Comparison of the phase separation and ssDNA condensation ability of RPA-Δ70N-eGFP in the presence and absence of p53. **(A)** The fluorescence images show that RPA-Δ70N-eGFP forms co-condensates with ssDNA in the presence and absence of p53. Scale bar, 5 μm. **(B)** Statistical analysis of condensate sizes for RPA-Δ70N-eGFP in the presence or absence of p53. **(C)** Representative images of RPA-eGFP-bound ssDNA after relaxation with increasing p53 concentrations. The distribution of the RPA-eGFP signal along the ssDNA is shown under each image. **(D)** Representative force – extension curves of RPA-bound ssDNA after 30 s relaxation in the presence of 20 nM RPA and varying concentrations of p53 (0

nM, 4 nM, or 20 nM). The grey curve represents the force−extension curve of naked ssDNA. **(E)** The normalized ssDNA extension (L/L_0) under different forces in the presence of 0 nM (n = 6), 4 nM (n = 9), and 20 nM (n = 8) p53. L_0 represents the counter length of the naked ssDNA. Data are shown as mean ± SD. With increasing p53 concentration, the condensed ssDNA by RPA can be reversed by high forces, indicating that p53 can weaken the stability of the RPA–ssDNA co-condensate.

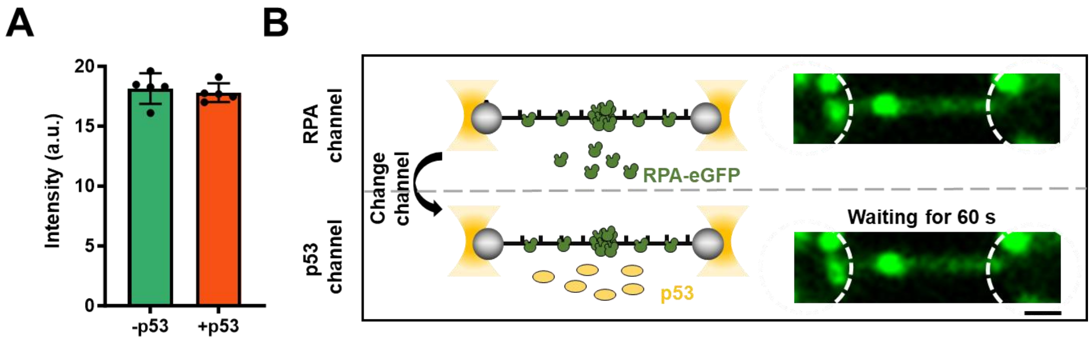

Supplementary Figure S10. RPA-eGFP binding on ssDNA is not impaired by p53. (A) The normalized intensities of RPA-eGFP (20 nM) on ssDNA in the presence (red) and absence (green) of p53 are comparable, suggesting that RPA retains its ssDNA binding ability. **(B)** RPA–ssDNA co-condensate was formed in the RPA channel (top) and then was moved to the p53 (20 nM) channel (bottom). After 60 s, the condensate still existed, indicating that p53 cannot dissolve the preformed RPA condensate.

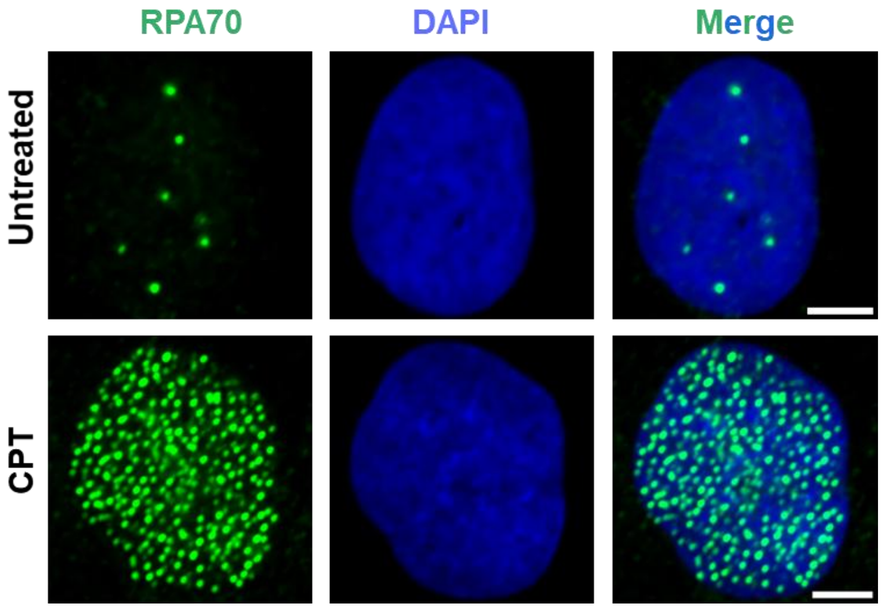

Supplementary Figure S11. Nuclear RPA condensates formed in U2OS cells after inducing DSBs. U2OS cells were treated with the DNA-damaging agent CPT (4 μM). After fixation and permeabilization, cells were incubated with an anti-RPA1 (green) antibody. After treatment with CPT, there is a significant increase in the number of RPA puncta localized in the nucleus compared to untreated cells. Nuclei were stained with DAPI (blue). Scale bars, 5 μm.

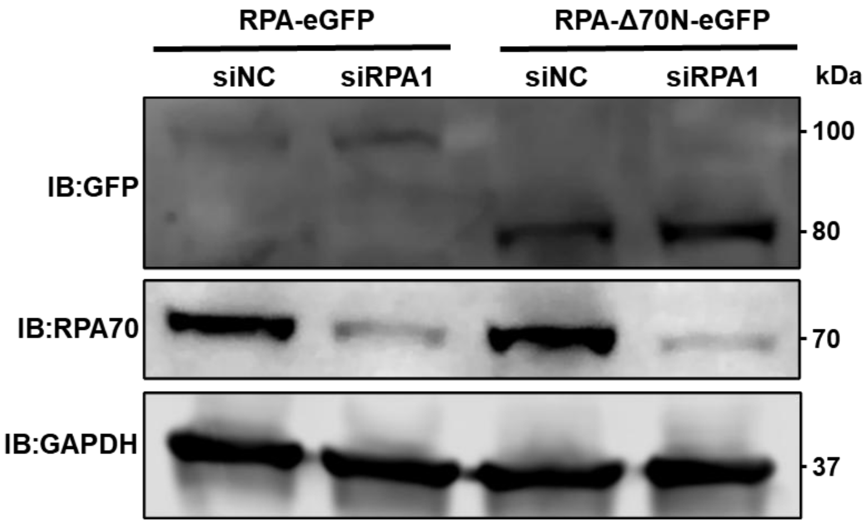

Supplementary Figure S12. The detection of RPA-eGFP and RPA-Δ70N-eGFP in U2OS cells. Immunoblotting analysis of RPA-eGFP, RPA-Δ70N-eGFP, and endogenous RPA70 in U2OS cells transfected with control siRNA (siNC) or a siRNA oligonucleotide targeting endogenous RPA70 (siRPA70). GAPDH served as the loading control.

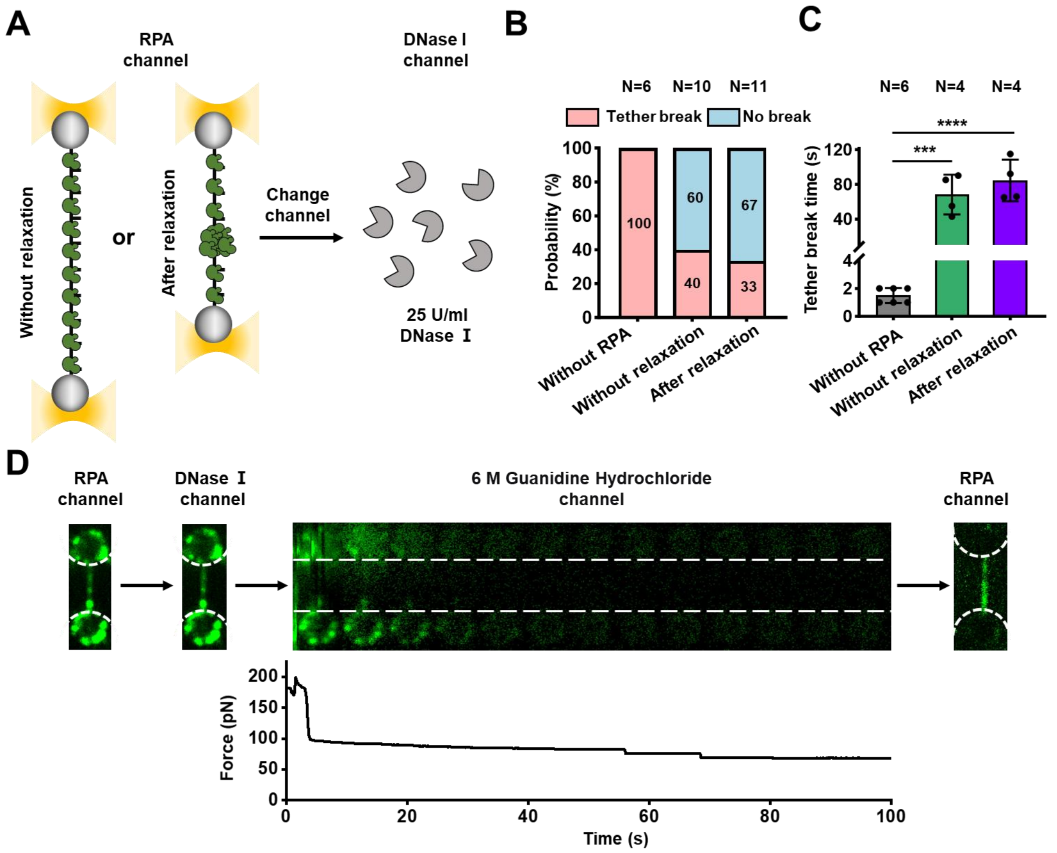

Supplementary Figure S13. The RPA-bound ssDNA with or without condensates digested with DNase I. **(A)** Schematic of the experimental procedure. RPA-bound ssDNA in the presence or absence of condensates was transferred to the DNase I channel. **(B)** Percentages of tether breakage and no breakage in the DNase I channel over 120 seconds. **(C)** Quantification of tether break time under different conditions. **(D)** Representative kymographs showing RPA-bound ssDNA with condensates incubated with guanidine hydrochloride to dissolve condensates, following incubation of RPA–ssDNA co–condensates in the DNase I channel for 120 seconds.

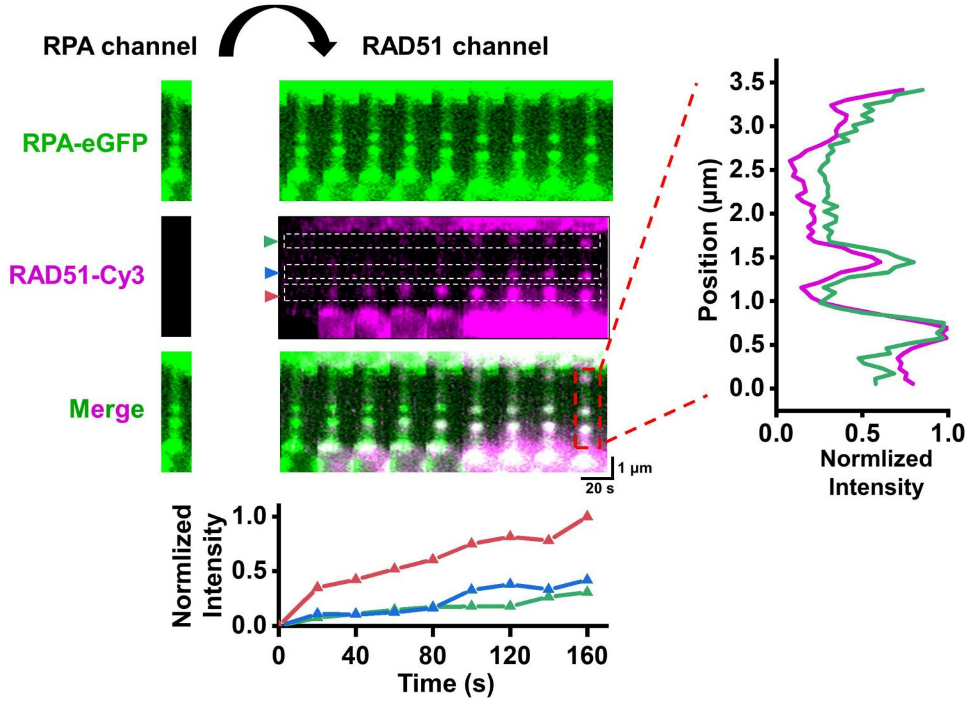

Supplementary Figure S14. RPA-ssDNA co-condensates recruit RAD51. Kymographs show that RAD51 specifically binds onto the RPA–ssDNA co-condensates in the RAD51 channel (1 μM). The change of the RAD51 signal is shown below the kymographs. The intensity profiles of the last frame in the fluorescence images are displayed on the right.

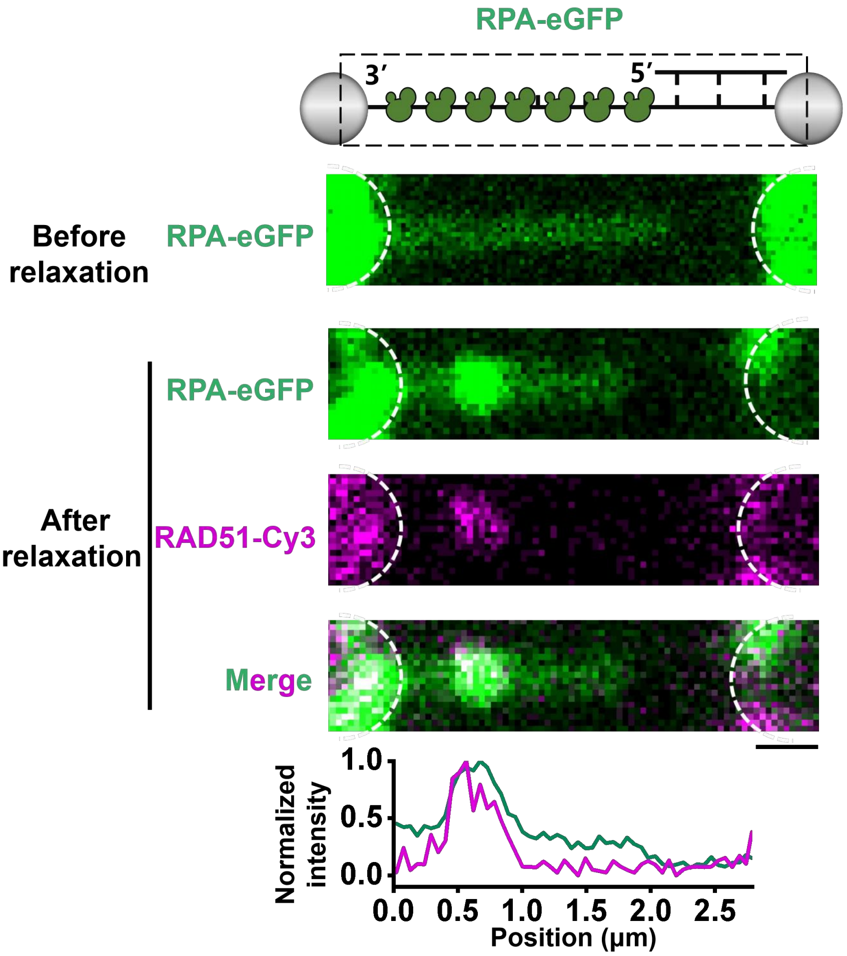

Supplementary Figure S15. The RPA–ssDNA co-condensates promote RAD51 recruitment on ss/dsDNA hybrid substrates. Representative kymographs showing RAD51-Cy3 (1 μM) binding to RPA-FL-eGFP-coated ssDNA (10.3 knt) under conditions with or without relaxation. The corresponding fluorescence intensity profiles of the analyzed molecules are shown below the kymographs.

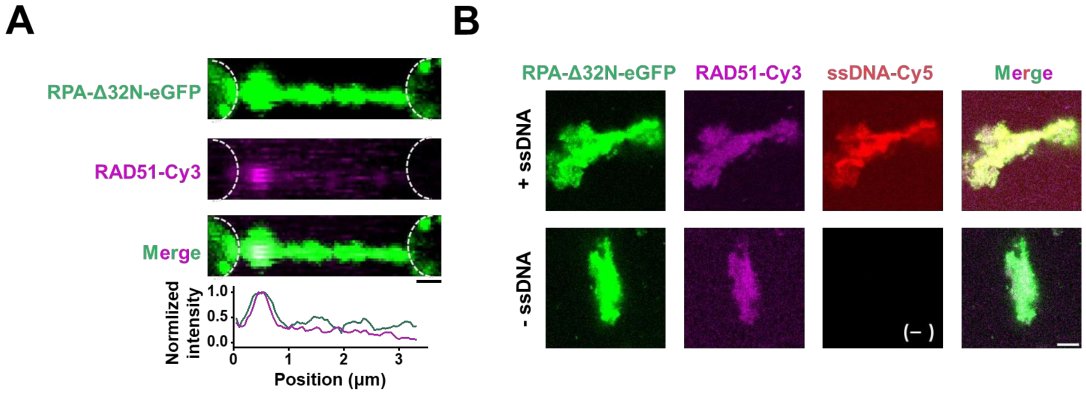

Supplementary Figure S16. RAD51 binds to RPA-Δ32N-eGFP aggregates. **(A)** Representative kymographs show that after ssDNA relaxation, RAD51-Cy3 (1 μM) binds to RPA-Δ32N-eGFP and ssDNA aggregates. The corresponding fluorescence intensity profiles of the examined molecules are displayed below the kymographs. **(B)** Representative fluorescence images of 10 μM RPA-Δ32N-eGFP droplets in the presence and absence of 1.7 μM 33-nt ssDNA-Cy5. Scale bar, 5 μm. RAD51 can also associate with irregular RPA-Δ32N aggregates.

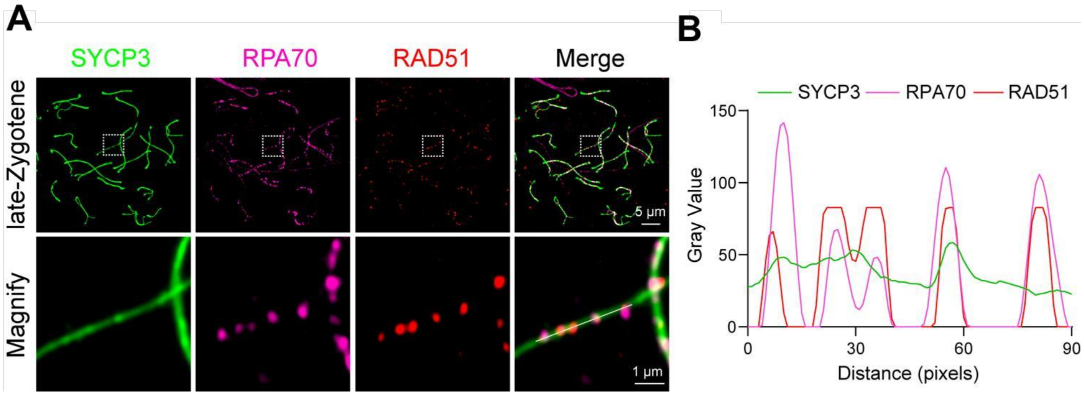

Supplementary Figure S17. RPA exhibits co-localization with RAD51 at meiotic DSB sites. **(A)** A representative immunofluorescence image of spermatocyte chromosomal spreads stained for SYCP3 (green), RPA1 (magenta), and RAD51 (red). **(B)** Intensity profiles of SYCP3, RPA, and RAD51 signals along the chromosomal axis (white line in panel a).

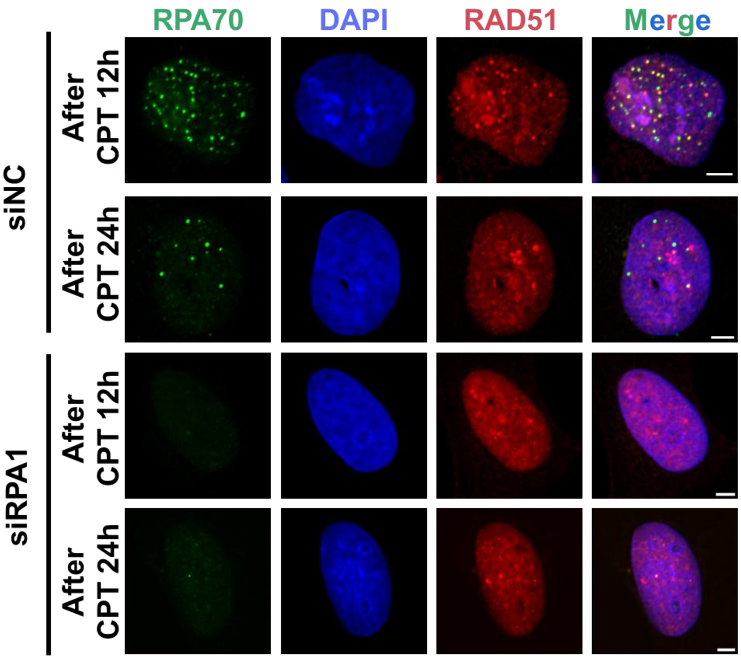

Supplementary Figure S18. Downregulation of RPA70 impairs RAD51 puncta formation. The knockdown of RPA70 impaired the recruitment of RAD51 to DNA damage sites in U2OS cells. Immunofluorescence of RPA (green) and RAD51 (red) in wild-type and RPA70 knockdown U2OS cells was allowed to recover from CPT treatment for 12 and 24 hours. Nuclei were stained with DAPI (blue). Scale bars, 5 μm.

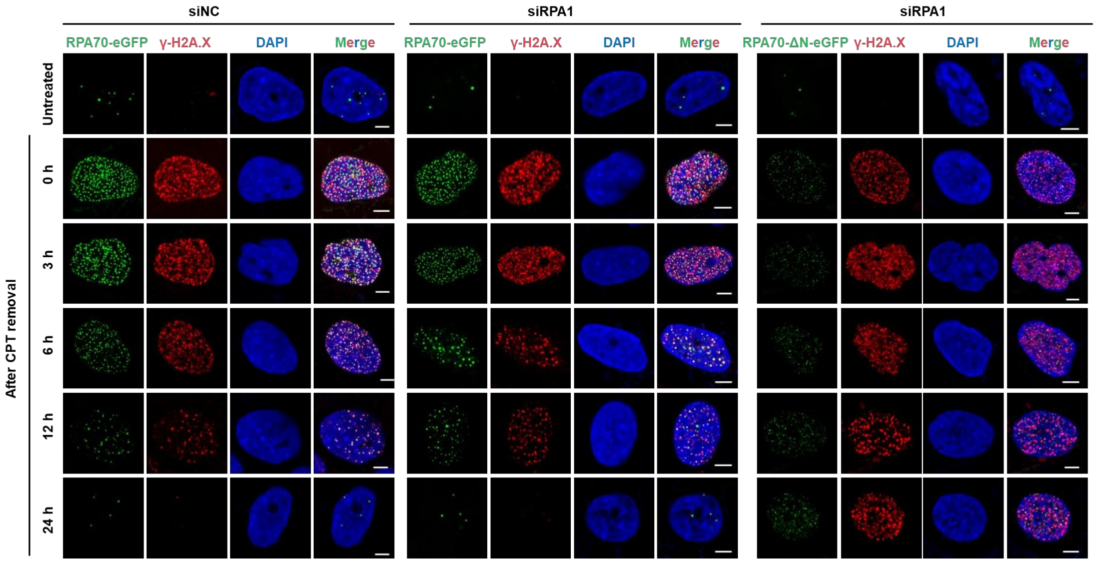

Supplementary Figure S19. RPA70-ΔN impairs the DSBs repairs under RPA1 knockdown conditions. Representative images of U2OS cells under CPT treatment, with or without RPA1 knockdown, showing the immunofluorescence of RPA70-eGFP/RPA-Δ70N-eGFP and γH2A.X. Following CPT treatment in RPA1-knockdown cells, those expressing exogenous RPA70-eGFP exhibited pan-nuclear γH2A.X staining, which progressively diminished over time and returned to pre-damage levels within 24 h, indicating successful repair. In stark contrast, RPA1 knockdown cells expressing RPA70-ΔN-eGFP showed a persistent γH2A.X signal that failed to resolve, demonstrating defective DSB repair.

Supplementary Table S1. Sequences of primers for the construction of the RNA–DNA hybrid templates.

Template	Name	Sequence
7.4-kbp dsDNA	forward	5′-AGGTTGTAGGCTCAAGAGGGTGTGTCC-3′
	reverse	5′-TGATAAGCAGAATGGCATCGTTCC-3′
7.4-kbp dsDNA for transcription	forward	5′-TAATACGACTCACTATAGGAGGTTGTAGGCTCAAGAG GGTGTGTCC-3′
	reverse	5′-TGATAAGCAGAATGGCATCGTTCC-3′
7.4-knt ssDNA	forward	5′-biotin-TGATAAGCAGAATGGCATCGTTCC-3′
12.3-kbp dsDNA	forward	5′-AGCCTTTGCCTCGCTATACA-3′
	reverse	5′-CAGCATAAGCGGCTACATGA-3′
12.3-kbp dsDNA for transcription	forward	5′-TAATACGACTCACTATAGGAGCCTTTGCCTCGCTATACA-3′
	reverse	5′-CAGCATAAGCGGCTACATGA-3′
12.3-knt ssDNA	forward	5′-biotin-CAGCATAAGCGGCTACATGA-3′
12.3-kbp ss/dsDNA for transcription	forward	5′-TAATACGACTCACTATAGGCGCCACTTCAGCACGAGATG-3′
	reverse	5′-CATGACGACTGGGGATTTGACGC-3′
12.3-kbp ss/dsDNA for 2.3-knt ssDNA	forward	5′-P-CATTGCCTGCTCTGCCGCT-3′
	reverse	5′-TGATAAGCAGAATGGCATCGTTCC-3′
12.3-kbp ss/dsDNA for 12.3-knt ssDNA	forward	5′-TAATACGACTCACTATAGGCGCCACTTCAGCACGAGATG-3′
	reverse	5′-biotin-TGATAAGCAGAATGGCATCGTTCC-3′

Supplementary Table S2. Sequences of siRNA duplexes for knockdown of endogenous RPA70.

Name	Sequence
siRPA70-S	5'-AGGCUUAUGGUGCUUCAAATT-3'
siRPA70-A	5'-UUUGAAGCACCAUAAGCCUTT-3'
SiNC-S	5'-UUCUCCGAACGUGUCACGUTT-3'
SiNC-A	5'-ACGUGACACGUUCGGAGAATT-3'
